# Accumulation of Linoleic Acid by Altered Peroxisome Proliferator-Activated Receptor-α Signaling Is Associated with Age-Dependent Hepatocarcinogenesis in *Ppara* Transgenic Mice

**DOI:** 10.3390/metabo13080936

**Published:** 2023-08-10

**Authors:** Xiaoyang Zhu, Qing Liu, Andrew D. Patterson, Arun K. Sharma, Shantu G. Amin, Samuel M. Cohen, Frank J. Gonzalez, Jeffrey M. Peters

**Affiliations:** 1 Department of Veterinary and Biomedical Science, The Center for Molecular Toxicology and Carcinogenesis, The Pennsylvania State University, University Park, State College, PA 16802, USA; lqing124@gmail.com (Q.L.); adp117@psu.edu (A.D.P.); jmp21@psu.edu (J.M.P.); 2 Department of Pharmacology, The Pennsylvania State University, Hershey, PA 17033, USA; aks14@psu.edu (A.K.S.); sga3@psu.edu (S.G.A.); 3 Department of Pathology and Microbiology, University of Nebraska Medical Center, Omaha, NE 68198, USA; scohen@unmc.edu; 4 Laboratory of Metabolism, National Cancer Institute, Bethesda, MD 20892, USA; gonzalef@mail.nih.gov

**Keywords:** peroxisome proliferator-activated receptor-α (PPARα), hepatocarcinogenesis, linoleic acid, CD4^+^ T cell, senescence

## Abstract

Long-term ligand activation of PPARα in mice causes hepatocarcinogenesis through a mechanism that requires functional PPARα. However, hepatocarcinogenesis is diminished in both *Ppara*-null and *PPARA*-humanized mice, yet both lines develop age-related liver cancer independently of treatment with a PPARα agonist. Since PPARα is a master regulator of liver lipid metabolism in the liver, lipidomic analyses were carried out in wild-type, *Ppara*-null, and *PPARA*-humanized mice treated with and without the potent agonist GW7647. The levels of hepatic linoleic acid in *Ppara*-null and *PPARA*-humanized mice were markedly higher compared to wild-type controls, along with overall fatty liver. The number of liver CD4^+^ T cells was also lower in *Ppara*-null and *PPARA*-humanized mice and was negatively correlated with the elevated linoleic acid. Moreover, more senescent hepatocytes and lower serum TNFα and IFNγ levels were observed in *Ppara*-null and *PPARA*-humanized mice with age. These studies suggest a new role for PPARα in age-associated hepatocarcinogenesis due to altered lipid metabolism in *Ppara*-null and *PPARA*-humanized mice and the accumulation of linoleic acid as part of an overall fatty liver that is associated with loss of CD4^+^ T cells in the liver in both transgenic models. Since fatty liver is a known causal risk factor for liver cancer, *Ppara*-null and *PPARA*-humanized mice are valuable models for examining the mechanisms of PPARα and age-dependent hepatocarcinogenesis.

## 1. Introduction

The peroxisome proliferator-activated receptors (PPARs) are members of the steroid/nuclear receptor family. PPARs have essential roles in cellular homeostasis, differentiation, inflammation, and modulation of carcinogenesis [1,2]. PPARα, one of three PPAR subtypes (PPARα, PPARβ/δ, and PPARγ) identified in 1990 [3,4] has important roles in the transport and oxidation of fatty acids in many tissues including the liver, heart, intestine, and kidney where lipids can be used as an energy source [5,6]. PPARs are ligand-activated transcription factors that dynamically regulate gene expression in response to endogenous or exogenous ligands. Ligands bind to PPAR, causing structural changes in the protein structure releasing co-factors that maintain a tight complex on chromatin and recruit other proteins to the PPAR complex to facilitate the transcription of target genes. Importantly, nuclear receptors such as PPARs exist in dynamic equilibrium between chromatin binding and an intracellular PPAR complex, due to the presence/absence of ligands, accessory proteins, and PPARs [7,8]. It is important to note that PPARα is the molecular target that mediates the lipid lowering effects of the widely prescribed fibrate class of hypolipidemic drugs [9]. The mechanism by which PPARα causes a decrease in serum triglycerides by fibrates is primarily through increased expression of PPARα target genes that facilitate the transport of fatty acids across membranes and catabolism in tissues that generates cellular ATP via oxidation of long-chain fatty acids and effectively decreases the triglycerides in serum [9,10,11]. Since most organisms typically exist in a fasting state where fatty acids are higher and able to bind with PPARα, this nuclear receptor is essential for regulating lipid homeostasis [12].

While it is clear that PPARα activation is essential for cellular energy homeostasis [9,10,11], pharmacological activation of the PPARα with synthetic ligands causes hepatocarcinogenesis in rodents through a mechanism that requires PPARα. Whereas wild-type mice which were fed a diet with the synthetic PPARα ligands Wy-14,643, bezafibrate, or GW7647 all exhibited close to 100% incidence of liver cancer after approximately one year of treatment, *Ppara*-null mice are largely refractory to this effect [13,14,15,16,17]. The mechanisms that mediate this are caused by PPARα-dependent down-regulation of let7c microRNA and E-cadherin/cadherin 1 (CDH1) in the liver that leads to higher expression of proteins that promote hepatocyte proliferation of cells with DNA damage [18,19,20]. Hepatocarcinogenesis is also diminished in *PPARA*-humanized mice after chronic treatment with either Wy-14,643 or GW7647 [13,14,16]. Results from studies examining the mechanism of PPARα-dependent liver cancer also show that activation of this receptor effectively decreases serum lipids in both rodents and humans, but the changes in the let7c pathway occur only in wild-type mice, which is not found in *PPARA*-humanized mice after chronic treatment with either Wy-14,643 or GW7647 [13,14,16]. In addition to these studies, there is other strong evidence indicating that a species difference exists between rodents and humans with regard to their susceptibility to liver cancer from PPARα ligands as rodents develop liver cancer but there is no epidemiological evidence that this occurs in humans [5,9,21,22]. Furthermore, increased hepatocellular proliferation, a key event in the mode of action of PPARα-induced hepatocarcinogenesis in rodents, does not occur in humans, as demonstrated in the chimeric humanized mouse liver model [23].

Studies using *Ppara*-null and *PPARA*-humanized mice demonstrated an essential role for PPARα in rodent liver cancer caused by PPARα ligands. However, some studies have revealed that spontaneous liver tumors develop in aged *Ppara*-null and *PPARA*-humanized mice in the absence of exogenous PPARα ligand administration [13,14,15,24]. Given the critical role of PPARα in regulating lipid catabolism, it is not surprising that *Ppara*-null mice exhibit reduced constitutive expression of hepatic lipid metabolizing enzymes [25]. Liver steatosis is found in aging *Ppara*-null mice [13,14], and hepatic steatosis is well-known to be a form of hepatocellular toxicity, leading to liver inflammation and increased incidences of hepatocellular tumors in rodents and in humans [26,27,28]. For these reasons, the present studies were designed to test the hypothesis that alterations in hepatic lipid metabolism cause liver cancer independent of PPARα. 

## 2. Materials and Methods

### 2.1. GW7647 Synthesis

2-Methyl-2-[[4-[2-[[(cyclohexylamino)carbonyl](4-cyclohexylbutyl)amino]ethyl]phenyl]thio]-propanoic acid (GW7647) was synthesized by the Penn State Cancer Institute Organic Synthesis Shared Resource as described previously [13,14,29]. The GW7647 synthesized was between 96.6 and 98.4% pure based on HPLC analyses.

### 2.2. Diets

Pelleted mouse chow was prepared (Dyets Inc., Bethlehem, PA, USA) containing either 0.0 (control) or 0.01% GW7647 and it was provided to mice *ad libitum*. The concentration of GW7647 was based on results showing that relative to controls, 0.01% GW7647 caused a similar increase in liver weight and hepatic expression of the PPARα target gene *cytochrome P450 4a10* (*Cyp4a10*) compared to 0.1% WY-14,643 after seven days of treatment [13,14]. Tap water was available *ad libitum.*

### 2.3. Study Designs

The tissue samples used for these studies were obtained from two previously published studies approved by the Pennsylvania State University Institutional Animal Care and Use Committee (IACUC31363, 17 May 2011) [13,14]. Briefly, both studies used wild-type, *Ppara*-null, or *PPARA*-humanized mice on the 129/Sv genetic background as previously described [30,31]. Two exposure paradigms were used (Figure S1). The first paradigm used six- to twelve-week-old male mice, either wild-type, *Ppara*-null or *PPARA*-humanized [13,14]. Ligand activation of PPARα was achieved by feeding the either control or 0.01% GW7647 diet to the three different lines of mice for either 26 weeks or ~75 weeks (Figure S1a). This group is referred to as “adult only”. For the second study, ligand activation of PPARα was initiated in perinatal mice on postnatal day 3 and continued into adulthood (Figure S1b). Neonatal mice were gavaged daily with a dose of 10 mg GW7647/body weight (kilogram) from postnatal day 3 to postnatal day 21, when the mice were weaned. After postnatal day 21, the mice were fed either the control diet or one containing 0.01% GW7647 ad libitum like the adult mice for either 26 weeks or ~75 weeks (Figure S1b). This group is referred to as “perinatal + adult”. Exposure to chemicals during prenatal and perinatal development can alter the developing conceptus differentially compared to adults, and may cause effects that persist/manifest later in life including cancer [32]. Ligand activation of PPARα in fetuses by clofibrate in pregnant rats causes peroxisome proliferation but younger fetuses appeared less responsive to this effect compared to fetuses exposed to clofibrate during later gestation and other studies have also noted that developing fetuses or neonates can exhibit varying levels of peroxisome proliferation and/or induction of hepatic PPARα target genes during perinatal development [33,34,35,36]. For these reasons, the present studies examined the effect of long-term administration of a PPARα agonist GW7647, on hepatocarcinogenesis using wild-type, *Ppara*-null, and *PPARA*-humanized mice with exposure beginning either in adult mice or those at the age of postnatal day 3. Importantly, these treatment paradigms were used to allow direct comparison between the changes in the liver metabolome in the present studies and the relative incidence of liver cancer induced in the presence or absence of a functional mouse or human PPARα noted in previous studies [13,14].

### 2.4. Hepatic Metabolomics Using 1H Nuclear Magnetic Resonance (NMR) and Data Analyses

Approximately fifty milligrams of liver tissue from mice were homogenized with a BeadBlaster Homogenizer (Southern Labware, Cumming, GA, USA) for 90 s (with a 5 s interval per 30 s) in microcentrifuge tubes containing zirconia/silica beads and pre-cooled chloroform and methanol (2:1) mixture. After one 90 s cycle, the liver tissue samples were cooled on ice for 15 min, and this procedure was repeated one more time. Samples were then centrifuged at 12,000× *g*, 4 °C for 10 min and the supernatants containing the lipid extracts were transferred to new tubes. The lipid extracts were dried with a speed vacuum and resuspended in deuterated chloroform (CDCl_3_, 0.03% (*v*/*v*) tetramethylsilane (TMS); Sigma-Aldrich, St Louis, MO, USA) and then transferred into NMR tubes.

^1^H NMR spectra of liver lipid extracts were acquired at 298 K using a Bruker AVIII 600 MHz NMR spectrometer (Bruker Biospin, Rheinstetten, BW, Germany) with a cryogenic probe, operating at a proton frequency of 600.13 MHz. The standard Bruker zg pulse sequence was employed for one-dimensional NMR spectra. Two-dimensional NMR spectra (^1^H-^1^H COSY and TOCSY, ^1^H-^13^C HSQC, and HMBC) were acquired to assist with the spectral assignment in addition to comparing these data with published databases [37,38,39,40].

^1^H NMR spectra were manually corrected for phase and baseline deformation using Topspin software (V3.5, Bruker Biospin, Rheinstetten, BW, Germany). The spectra of liver lipid extracts were referenced to the TMS peak (δ, 0 ppm). The spectra ranging from δ 0.5 to δ 5.5 ppm were integrated with an equal width of 0.002 ppm, using the AMIX package (V3.8, Bruker Biospin, Rheinstetten, BW, Germany). Areas of identified lipid peaks were normalized to wet weight of liver tissues. The relative amount of hepatic lipid for the groups with different treatments was normalized to wild-type control and represents the fold change. Values represent the mean ± S.E.M.

### 2.5. Hepatic Fatty Acid Profiling Using Gas Chromatography Coupled Mass Spectrometry (GC-MS)

GC-MS was used as previously described (Zhang, Toxicology, 2020). Briefly, ten milligrams of liver tissue were homogenized with a BeadBlaster Homogenizer (Southern Labware, Cumming, GA, USA) for 90 s (with a 5 s interval for 30 s) in microcentrifuge tubes containing zirconia/silica beads with pre-cooled methanol (HPLC grade). After one 90 s cycle, the liver samples were cooled on ice for 15 min, and this procedure was repeated twice. The liver homogenate was put into glass conical centrifuge tubes and the fatty acids were methylated using a previously reported method with minor modification [41]. An internal standard (1 mg/mL methyl heptadecanoate, 0.5 mg/mL methyl tricosanoate, and 2 mg/mL butylated hydroxytoluene in hexane) was added into each tube containing liver homogenate, followed by the addition of 1 mL methanol/hexane mixture (4:1) and vortexing. Tubes were cooled in liquid nitrogen and then pre-cooled acetyl chloride was added into the mixture slowly. After submersion in liquid nitrogen, the samples were placed at room temperature in the dark for twenty-four hours. Samples were cooled on ice and neutralized by slowly adding 6% K_2_CO_3_ hexane to the samples, then they were vortexed, and centrifuged to obtain the supernatant. The extraction procedure was repeated three times and combined for each sample. The supernatant in the tubes were then placed in a fume hood to remove the organic solvent. These lipid samples were reconstituted with hexane and transferred to autosampler vials. The fatty acids were quantified using an Agilent 7890A-5975C GC-MS system (Agilent Technologies, Santa Clara, CA, USA) with a previously described procedure [42]. A Supelco 37 Component FAME Mix (Sigma-Aldrich, St Louis, MO, USA) was used to help identify GC-MS peaks. 

Detected and identified fatty acids were quantified by comparing integrated peak areas following normalization to the internal standards. The results were expressed as microgram fatty acids per milligram of liver tissue as the mean ± standard deviation.

### 2.6. Quantitative Western Blot Analyses of Hepatic 4-Hydroxynonenal (4-HNE)

Approximately fifty milligrams of liver tissue were homogenized with a Dounce tissue grinder in a tube containing RIPA lysis buffer (50 mM Tris base, 150 mM sodium chloride, 1% triton, 0.5% SDS, 0.5% sodium deoxycholate, pH 8.6, and protease inhibitors) on ice. The liver homogenate was centrifuged at 10,000× *g*, 4 °C for 10 min. The supernatant was collected and used for analyses. Quantitative western blotting was performed to measure the level of 4-hydroxynonenal (4-HNE) in the liver as previously described [43]. Briefly, fifty micrograms of protein from each sample were resolved using SDS-PAGE. The gel samples were transferred onto a polyvinylidene difluoride membrane with electroblotting. After blocking the membrane with Tris-buffered saline with 5% milk, the membrane was incubated overnight at 4 °C with an anti-HNE primary antibody (Abcam, ab46545, Waltham, MA, USA) followed by incubation with a biotinylated secondary antibody. Immunoreactive proteins were detected after incubation in [^125^I]-labeled streptavidin using phosphorimaging analysis. Hybridization signals for 4-HNE were normalized to the hybridization signal of the housekeeping protein; lactate dehydrogenase (LDH) (Rockland, Gilbertsville, PA, USA). Independent triplicate samples were used for the analysis of each treatment group. 

### 2.7. Thiobarbituric Acid Reactive Substance (TBARS) Assay

The TBARS assay was performed using a previously reported method with modification [44]. Briefly, twenty milligrams of liver tissue were homogenized with a BeadBlaster Homogenizer (Southern Labware, Cumming, GA, USA) for 90 s (with a 5 s interval for 30 s) in Eppendorf tubes containing zirconia/silica beads with pre-cooled RIPA buffer (with protease inhibitor). After one 90 s cycle, the liver samples were cooled on ice for 15 min, and this procedure was repeated two more times. The liver homogenate was centrifuged at 3000× *g*, 4 °C for 10 min. The supernatants were transferred to a new tube and then ice cold 10% trichloroacetic acid was added to precipitate proteins. These samples were incubated on ice for 15 min and then centrifuged again at 2200× *g*, 4 °C for 15 min. The supernatant and prepared standards were transferred to new tubes, and an equal volume of 0.67% (*w*/*v*) thiobarbituric acid was added. These samples were then placed in a boiling water bath for 10 min and cooled down after at room temperature for 30 min. Duplicate samples and standards were transferred into a clear 96-well plate and the absorbance at 532 nm was measured to determine the amount of TBARS in each sample. 

### 2.8. Immunohistochemistry

Formalin-fixed, paraffin-embedded liver tissue sections on slides (4 μm) were placed in a 60 °C oven for 30 min. Slides were deparaffinized, washed in decreasing amounts of ethanol (90–70%), and rehydrated after washing with distilled water and then phosphate buffered saline (PBS). Rehydrated slides were immersed in buffer (10 mM Tris base, 1 mM EDTA (disodium salt), pH 9.0; Cold Spring Harbor Protocols) and subjected to heat-induced epitope retrieval with an IHCWORLD Steamer Set for 50 min. After the slides in buffer cooled down to ambient temperature, they were washed three times with 0.01 M PBS (pH 7.4) for 5 min. Fresh 3% H_2_O_2_ was applied to each slide section at room temperature for 10 min to inactivate endogenous peroxidase and was washed three times with 0.01 M PBS (pH 7.4) for 5 min each to remove residue of H_2_O_2_. Slides were blocked with 5% normal goat serum for 30 min at room temperature and washed three times. After blocking and washing, slides were incubated with either an anti-CD4 antibody (Abcam, ab183685, Waltham, MA, USA), an anti-p21 antibody (Abcam, ab188224, Waltham, MA, USA), or an anti-CDKN2A/p16INK4a antibody (Abcam, ab211542, Waltham, MA, USA) and placed into a humidifier at 4 °C overnight and then into a 37 °C incubator for 45 min. After three washes with 0.01 M PBS (pH 7.4) for 5 min, a horseradish peroxidase-conjugated secondary antibody (Abcam, ab214880, Waltham, MA, USA) was applied to each slide in the humidifier for one hour. After this incubation, the slides were washed three times with 0.01 M PBS (pH 7.4) for 5 min. Processed slides were then incubated with 3,3-diaminobenzidine (DAB) working solution (Vector Laboratories, Newark, CA, USA) at room temperature for one minute and then immersed into tap water to stop the reaction. Slides were sealed with mounting medium (Vector Laboratories, Newark, CA, USA). 

Four representative liver tissue slides were examined from each treatment group. For quantification of the cells that stained positive for each protein, ten fields were counted on each slide with a microscope (100×). Regions with a large blood vessel were not examined. The number of positive cells was quantified using ImageJ (Vx, NIH). The number of positive cells from each of the ten fields from four representative mice examined were used to calculate the average number of positive cells per mouse liver section. Relative positive cell number of groups with different treatments was normalized to wild-type control and represents the fold change. Values represent the mean ± S.E.M.

### 2.9. Serum IFNγ and TNFα

The concentrations of interferon gamma (IFNγ) and tumor necrosis factor alpha (TNFα) in serum were quantified by ELISA assays (BMS609, BMS607HS, Invitrogen, Vienna, Austria). Assays were performed using the manufacturer’s recommended protocols.

### 2.10. Statistical Analyses

The data were analyzed by analysis of variance followed by Tukey’s multiple comparisons test (Prism 9.0; GraphPad Software Inc., La Jolla, CA, USA). For all analyses, differences are described when *p* ≤ 0.05.

## 3. Results

To examine the role of mouse or human PPARα in modulating hepatic lipids, wild-type, *Ppara*-null, or *PPARA*-humanized mice were fed either a control diet or one containing 0.01% GW7647 for either 26 weeks or ~75 weeks (Figure S1). Two different exposure paradigms were used. In the first paradigm, dietary GW7647 was provided to adult mice for either 26 weeks or ~75 weeks (Figure S1a). In the second paradigm, dietary GW7647 was provided to perinatal mice through adulthood for either 26 weeks or ~75 weeks (Figure S1b). Since the focus of these studies is on the effect of constitutive expression/activity of PPARα and the effect of specific ligand activation of PPARα, the results are presented in this order, respectively. The effect of age on exposure was also examined by the two different paradigms as the age at termination was 6–12 weeks older in the mice after 26 weeks or ~75 weeks for the “adults only” group compared to the “perinatal + adult” group (Figure S1). Using these approaches allows for comparison with PPARα-dependent differences in liver carcinogenesis previously reported [13,14].

### 3.1. Differential Alterations of Hepatic Lipids by Mouse and Human PPARα 

**Figure 1:**
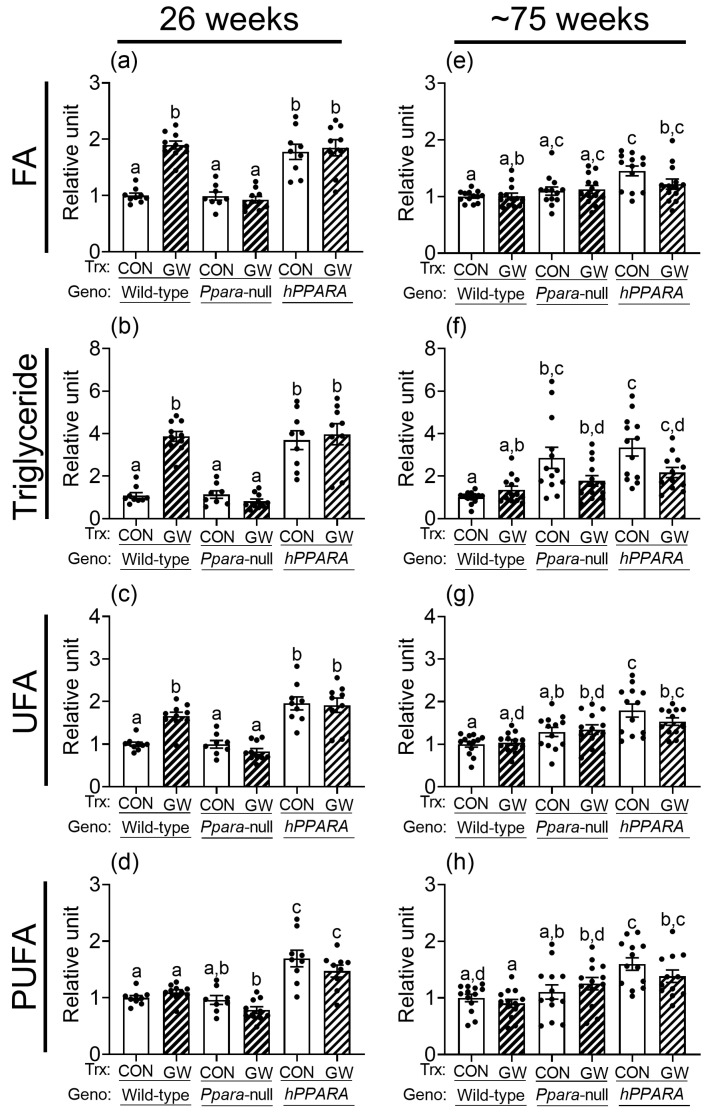
Relative levels of fatty acids (**a**,**e**), triglycerides (**b**,**f**), unsaturated fatty acids (UFA; **c**,**g**), or polyunsaturated fatty acids (PUFA; **d**,**h**) in lipophilic liver extracts from “adult only” groups of wild-type, *Ppara*-null, or *PPARA*-humanized mice with or without ligand activation of PPARα after 26 weeks (**a**–**d**) or ~75 weeks (**e**–**h**). Fatty acid residues (R-C**H**_3_), triglycerides (C_1_**H** and C_3_**H** of glycerol), unsaturated fatty acid (UFA) residues (-C**H**=C**H**-), and polyunsaturated fatty acid (PUFA) residues (-CH=CH-C**H**_2_-(CH=CH-CH_2_-)_n_). CON, control mouse group; GW, GW7647-treated mouse group. The relative average amount of lipids in the liver of mice with different treatments was normalized to wild-type control and represents the fold change. Values represent the mean ± S.E.M. Groups with different superscript letters are significantly different at *p* ≤ 0.05. (One-way ANOVA followed by Tukey’s multiple comparisons test).

Thirty signal peaks from lipids were identified by NMR in liver samples from both treatment paradigms (Figure S2, Tables S1–S4). Most of these were identified as fatty acids, cholesterols, glycerides, and phospholipids. After 26 weeks, the relative levels of total hepatic fatty acids, triglycerides, unsaturated fatty acids, and polyunsaturated fatty acids were not different between control wild-type or *Ppara*-null mice with either treatment paradigm (Figure 1 and Figure S3). Similarly, after ~75 weeks the relative amount of total hepatic fatty acids, unsaturated fatty acids, and polyunsaturated fatty acids were not different between control wild-type or *Ppara*-null mice with either treatment paradigm (Figure 1 and Figure S3). However, higher hepatic triglycerides were noted in control *Ppara*-null mice compared to control wild-type mice after ~75 weeks in the “adult-only” group (Figure 1). This effect was not noted in the similarly treated “perinatal + adult” group of control *Ppara*-null mice compared to control wild-type mice (Figure S3). The relative amount of total hepatic fatty acids, triglycerides, unsaturated fatty acids, and polyunsaturated fatty acids was higher in control *PPARA*-humanized mice compared to control wild-type mice after 26 or ~75 weeks in the “adult-only” and “perinatal + adult” groups (Figure 1 and Figure S3). 

**Figure 2:**
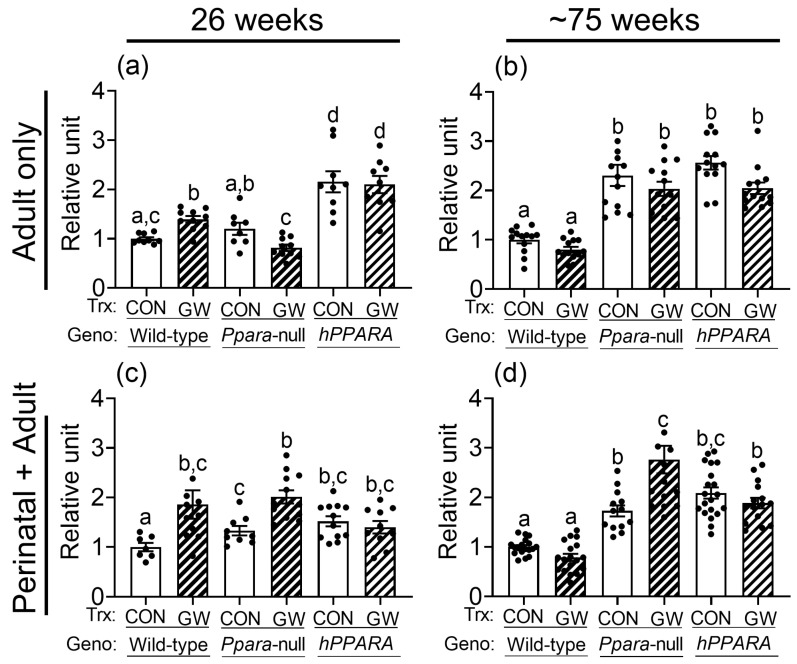
NMR detection of linoleic acid in lipophilic liver extracts from both “adult only” and “perinatal + adult” groups of wild-type, *Ppara*-null, or *PPARA*-humanized mice with or without ligand activation of PPARα. NMR was used to detect linoleic acid after either 26 weeks or ~75 weeks in “adult only” (**a**,**b**) or “perinatal + adult” (**c**,**d**) groups. Relative amount of lipids in livers was normalized to wild-type control and represents the fold change. Values represent the mean ± S.E.M. Groups with different superscript letters are significantly different at *p* ≤ 0.05. (One-way ANOVA followed by Tukey’s multiple comparisons test).

No differences in total hepatic omega 3 fatty acids or docosahexaenoic acid were noted between control or *Ppara*-null mice from either treatment paradigm (Figure S4). The average concentration of linoleic acid in the liver was higher in control *Ppara*-null mice and control *PPARA*-humanized mice compared to wild-type controls after 26 weeks or ~75 weeks in the “perinatal + adult” group (Figure 2c,d). Higher liver linoleic acid was only noted in control *Ppara*-null mice after ~75 weeks in the “adult only” group (Figure 2a,b). The average concentration of linoleic acid in the liver was higher in control *PPARA*-humanized mice compared to wild-type control for both treatment paradigms (Figure 2). The higher hepatic concentration of linoleic acid in control *Ppara*-null and control *PPARA*-humanized mice detected by NMR after ~75 weeks was confirmed with GC/MS (Figure S5). Interestingly, the average hepatic concentration of linoleic acid was higher in both *Ppara*-null and control *PPARA*-humanized mice compared to control wild-type mice after 26 or ~75 weeks for both treatment paradigms (Figure 2). 

### 3.2. Activation of Mouse or Human PPARα with a High Affinity Ligand Differentially Alters Hepatic Lipids

Ligand activation of PPARα with GW7647 in wild-type mice for 26 weeks and ~75 weeks altered the amount of total cholesterol, esterified cholesterol, free cholesterol, total fatty acids, monoglycerides, phophatidylethanolamine, sphingomyelin, phosphatidylcholine, total phospholipids, and diglycerol phosphate in the liver (Tables S1–S4). Ligand activation of PPARα with GW7647 for 26 weeks in wild-type mice caused an increase in hepatic fatty acids, triglycerides, and unsaturated fatty acids but no changes in polyunsaturated fatty acids, in both “adult only” and “perinatal + adult” groups (Figure 1 and Figure S3). This effect after 26 weeks was diminished in similarly treated *Ppara*-null mice in both the “adult only” and “perinatal + adult” groups (Figure 1 and Figure S3). Ligand activation of PPARα with GW7647 for 26 weeks in *PPARA*-humanized mice did not cause an increase in hepatic fatty acids, triglycerides, unsaturated fatty acids, or polyunsaturated fatty acids in the “perinatal + adult ” group compared to wild-type controls (Figure S3). However, it is important to note that in *PPARA*-humanized mice administered with GW7647 for 26 weeks, hepatic fatty acids, triglycerides, unsaturated fatty acids, or polyunsaturated fatty acids in the “adult only” control and ligand-treated groups were high compared to wild-type control but had similar levels as those observed after ligand activation of PPARα in wild-type mice (Figure 1). A similar trend was observed in *PPARA*-humanized mice administered GW7647 for 26 weeks in the “perinatal + adult” group compared to wild-type controls (Figure S3). After ~75 weeks, no ligand-dependent increases in hepatic fatty acids, triglycerides, and unsaturated fatty acids were observed in any genotype, in either treatment paradigms (Figure 1 and Figure S3). It is important to note that compared to wild-type controls, liver triglycerides, unsaturated fatty acids, and polyunsaturated fatty acids were relatively higher in *PPARA*-humanized mice after ~75 weeks of ligand activation of PPARα by GW7647 in the “adult only” group, but not in the “perinatal + adult” group (Figure 1 and Figure S3).

Ligand activation of PPARα with GW7647 in wild-type mice caused no change in total hepatic omega 3 fatty acids or docosahexaenoic acid compared to respective wild-type control with either treatment paradigm (Figure S4). Notably, liver linoleic acid was higher in wild-type mice after 26 weeks of dietary GW7647 (Figure 2a,c), but not after ~75 weeks, with both treatment paradigms (Figure 2b,d). In *Ppara*-null mice, administration of dietary GW7647 did not affect liver omega 3 fatty acid concentrations compared to controls after 26 or ~75 weeks in both the “adult only” group and “perinatal + adult” group (Figure S4). However, administration of dietary GW7647 to *Ppara*-null mice for either 26 or ~75 weeks resulted in higher liver docosahexaenoic acid in the “perinatal + adult” group compared to both wild-type and *Ppara*-null controls (Figure S4); this effect was not observed in the similarly treated “adult-only” group (Figure S4). Interestingly, dietary administration of GW7647 in *Ppara*-null mice also resulted in higher liver linoleic acid compared to both wild-type and *Ppara*-null controls, but this effect was only observed in the “perinatal + adult” group (Figure 2c), and not in the “adult only” group (Figure 2a). Administration of dietary GW7647 to *PPARA*-humanized mice for 26 weeks or ~75 weeks caused no change in liver omega 3 fatty acids or docosahexaenoic acid in the “adult only” and “perinatal + adult” groups compared to control (Figure S4). It is also important to note that the average level of liver omega 3 fatty acids or docosahexaenoic acid was higher in the “adult only” groups compared to wild-type control but dietary administration of GW7647 did not further alter these levels (Figure S4). Similarly, while the average concentration of hepatic linoleic acid was not affected by GW7647, liver linoleic acid levels were higher after 26 weeks or ~75 weeks in *PPARA*-humanized mice in the “adult only” and “perinatal + adult” groups compared to control (Figure 2). The NMR analyses described above were confirmed with GC/MS (Figure S5). The average concentrations of linoleic acid in liver were not altered by ligand activation of PPARα with GW7647 in wild-type mice ~75 weeks for both the “adult only” and “perinatal + adult” groups (Figure 2 and Figure S5). By contrast, the average concentration of hepatic linoleic acid was markedly higher in both the control and GW7647-treated *Ppara*-null or *PPARA*-humanized mice compared to control and GW7647-treated wild-type mice after ~75 weeks (Figure 2 and Figure S5).

### 3.3. Increased Lipid Peroxidation Is Not Associated with Hepatic Fatty Acid Accumulation

**Figure 3:**
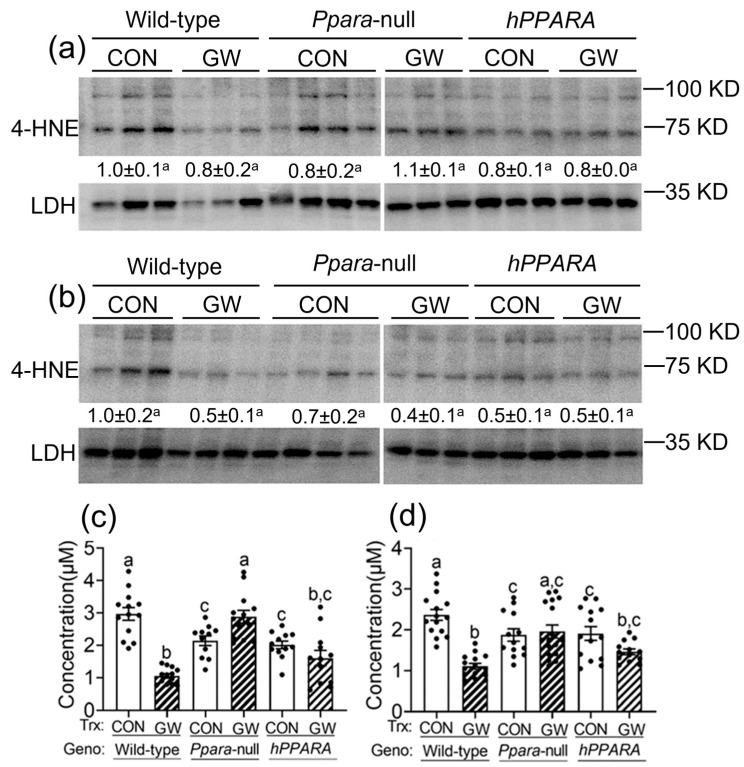
Assessment of oxidative stress in liver from “adult only” and “perinatal + adult” groups of wild-type, *Ppara*-null, or *PPARA*-humanized mice with or without ligand activation of PPARα. Both 4-hydroxynonenal (4-HNE, **a**,**b**) and malondialdehyde (MDA, **c**,**d**) levels in liver were measured after ~75 weeks in mice from both treatment paradigms. a and c, “adult only”; b and d, “perinatal + adult” groups. For the 4-HNE Western blot analyses (**a**,**b**) the relative amount of 4-HNE was normalized to the signal for lactate dehydrogenase (LDH) for wild-type control and represents the fold change. All values represent the mean ± S.E.M. Groups with different superscript letters are significantly different at *p* ≤ 0.05. (One-way ANOVA followed by Tukey’s multiple comparisons test).

Reactive oxygen species produced by lipid peroxidation can damage membrane structure/function, contribute to mutagenesis/carcinogenesis, and ultimately lead to cell death. To determine whether increased oxidative stress results from the observed accumulation of hepatic linoleic acid in *Ppara*-null or *PPARA*-humanized mice, 4-hydroxy-2-nonenal (4-HNE) and malondialdehyde (MDA) were measured (the main metabolites produced by peroxidation of linoleic acid). The average level of liver 4-HNE was not different between any genotype or treatment group, with either “adult only” and “perinatal + adult” groups compared to wild-type control (Figure 3a,b). In contrast, the hepatic concentration of MDA was lower in both control in *Ppara*-null or *PPARA*-humanized mice in either “adult only” and “perinatal + adult” groups compared to control wild-type mice (Figure 3c,d). Ligand activation of PPARα with GW7647 in wild-type mice in the “adult only” or “perinatal + adult” groups caused a decrease in liver MDA concentration compared to control (Figure 3c,d). This effect was not observed in similarly treated *Ppara*-null or *PPARA*-humanized mice with either treatment paradigm (Figure 3c,d). 

### 3.4. Hepatic CD4^+^ T Lymphocytes Is Inversely Correlated with Linoleic Acid Accumulation

**Figure 4:**
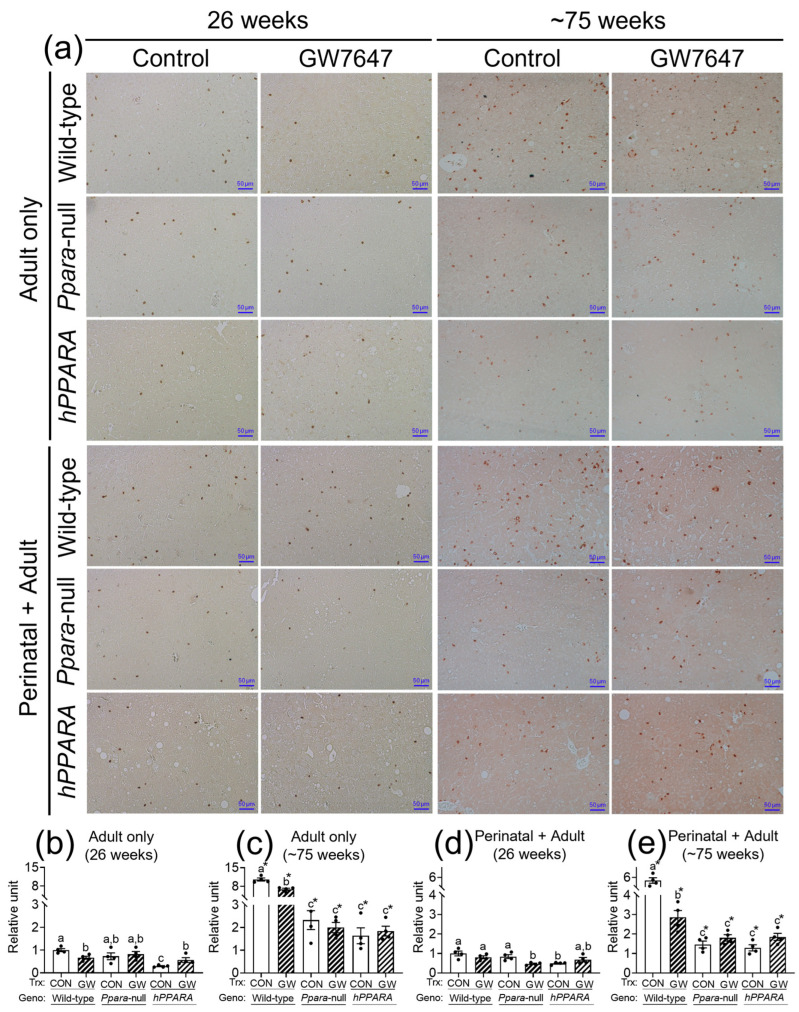
Representative photomicrographs of immunohistochemical detection of CD4+ T cells (**a**) in liver from “adult only” and “perinatal + adult” groups of wild-type, *Ppara*-null, or *PPARA*-humanized mice with or without ligand activation of PPARα with GW7647 after 26 weeks (**left panels**) or ~75 weeks (**right panels**). The relative amount of CD4^+^ T cells in liver was normalized to wild-type control and represents the fold change (**b**–**e**). Values represent the mean ± S.E.M. Groups with different superscript letters are significantly different at *p* ≤ 0.05. * significantly different at *p* ≤ 0.05 for the mouse treatment groups after 26 weeks (**b**,**d**) compared to the mouse treatment groups at ~75 weeks (**c**,**e**), respectively. (One-way ANOVA followed by Tukey’s multiple comparisons test).

The concentration of linoleic acid was higher in the liver of all the *Ppara*-null and humanized-*PPARA* mice with and without ligand activation of PPARα with GW7647. As previous studies showed that higher hepatic linoleic acid causes loss of CD4^+^ T lymphocytes and promotes hepatocarcinogenesis [45,46,47], the relative abundance of CD4^+^ T lymphocytes was measured by immunohistochemistry. In the “adult only” and “perinatal + adult” groups of control wild-type mice there were more hepatic CD4^+^ T lymphocytes after ~75 weeks compared to that observed after 26 weeks in their respective control (Figure 4a). In the “adult only” and “perinatal + adult” groups of control *Ppara*-null and *PPARA*-humanized mice there were also more CD4^+^ T lymphocytes in the liver after ~75 weeks compared to that observed after 26 weeks in their respective control (Figure 4). However, by contrast, the average number of liver CD4^+^ T lymphocytes in the “adult only” and “perinatal + adult” groups of control *Ppara*-null and control *PPARA*-humanized mice was lower compared to control wild-type mice from both treatment paradigms (Figure 4), particularly after ~75 weeks. Ligand activation of PPARα with GW7647 in wild-type mice in the “adult only” group caused a decrease in hepatic CD4^+^ T lymphocytes after 26 or ~75 weeks compared to the respective control group (Figure 4). Similarly, ligand activation of PPARα with GW7647 in wild-type mice in the “perinatal + adult” caused a decrease in hepatic CD4^+^ T lymphocytes after ~75 weeks (but not after 26 weeks) compared to the respective control (Figure 4). This decrease in liver CD4^+^ T lymphocytes by ligand activation of PPARα with GW7647 after 26 or ~75 weeks was not observed in either *Ppara*-null and *PPARA*-humanized mice (Figure 4). The lower number of liver CD4^+^ T cells correlated with higher concentrations of liver linoleic acid (Figure S6). 

### 3.5. Senescent Hepatocytes Increase in Liver of GW7647-Treated Mice 

**Figure 5:**
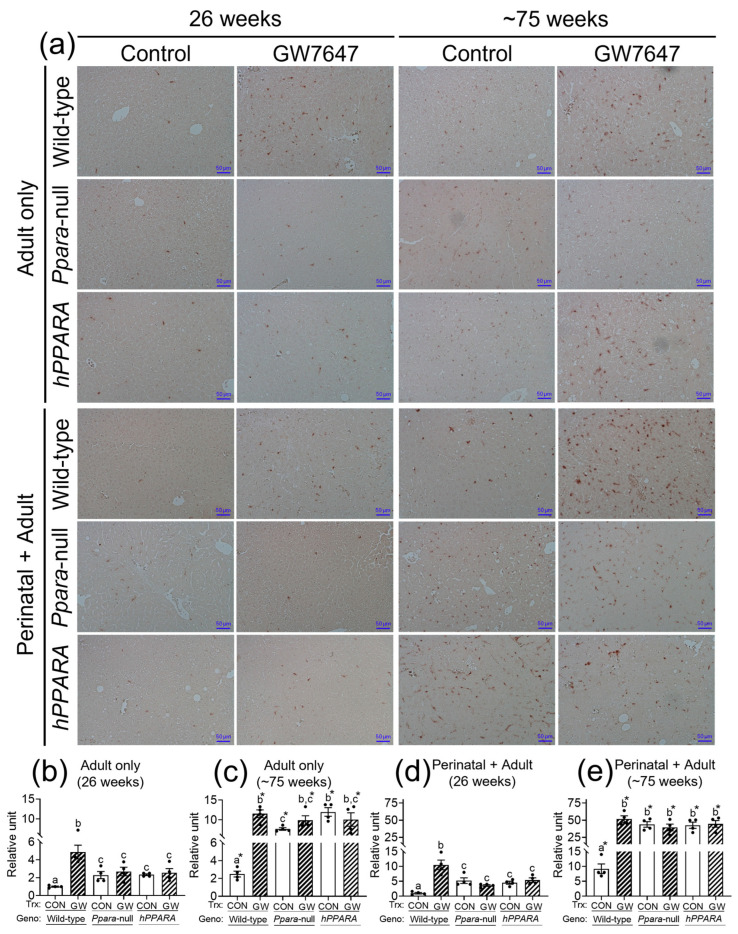
Representative photomicrographs of immunohistochemical detection of P16 (**a**) in liver from “adult only” and “perinatal + adult” groups of wild-type, *Ppara*-null, or *PPARA*-humanized mice with or without ligand activation of PPARα with GW7647 after 26 weeks (**left panels**) or ~75 weeks (**right panels**). The relative amount of P16-positive cells in liver was normalized to wild-type control and represents the fold change (**b**–**e**). Values represent the mean ± S.E.M. Groups with different superscript letters are significantly different at *p* ≤ 0.05. * significantly different at *p* ≤ 0.05 for the mouse treatment groups after 26 weeks (**b**,**d**) compared to the mouse treatment groups at ~75 weeks (**c**,**e**), respectively. (One-way ANOVA followed by Tukey’s multiple comparisons test).

**Figure 6:**
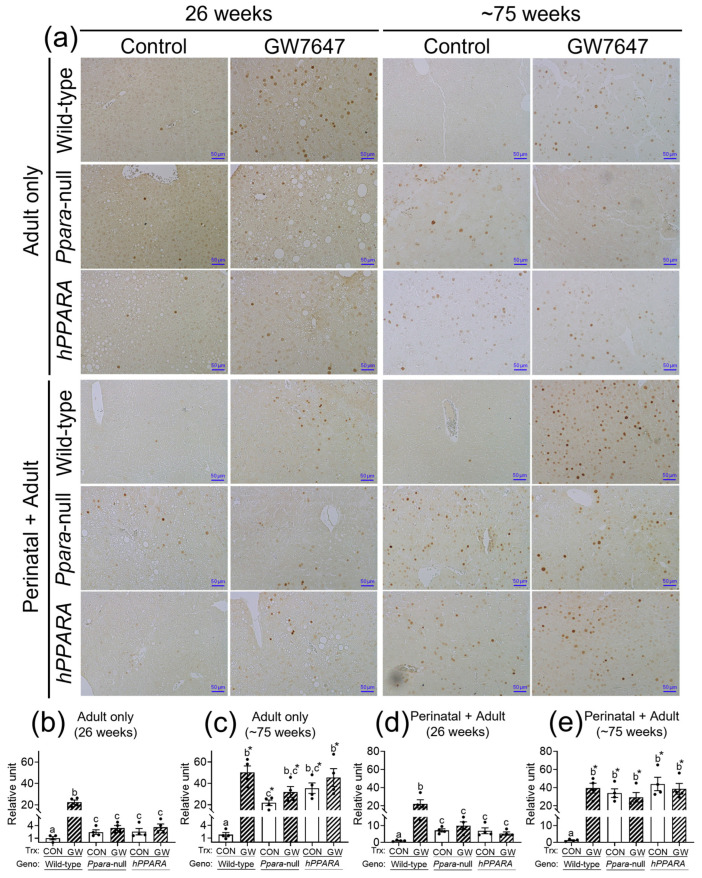
Representative photomicrographs of immunohistochemical detection of P21 (**a**) in liver from “adult only” and “perinatal + adult” groups of wild-type, *Ppara*-null, or *PPARA*-humanized mice with or without ligand activation of PPARα with GW7647 after 26 weeks (**left panels**) or ~75 weeks (**right panels**). Relative amount of P21-positive cells in liver was normalized to wild-type control and represents the fold change (**b**–**e**). Values represent the mean ± S.E.M. Groups with different superscript letters are significantly different at *p* ≤ 0.05. * significantly different at *p* ≤ 0.05 for the mouse treatment groups after 26 weeks (**b**,**d**) compared to the mouse treatment groups at ~75 weeks (**c**,**e**), respectively. (One-way ANOVA followed by Tukey’s multiple comparisons test).

CD4^+^ T lymphocytes have an important role in clearing senescent hepatocytes. Thus, we examined senescent markers to determine if linoleic acid associated liver CD4^+^ T lymphocyte loss influences senescence of hepatocytes. In the “adult only” and “perinatal + adult” groups of control wild-type mice there were few hepatocytes expressing P16 or P21 after 26 or ~75 weeks (Figure 5 and Figure 6). By contrast, in the “adult only” and “perinatal + adult” groups of control *Ppara*-null and *PPARA*-humanized mice there were more hepatocytes expressing P16 and P21 after 26 or ~75 weeks compared to wild-type controls (Figure 5 and Figure 6). Ligand activation of PPARα with GW7647 in wild-type mice in the “adult only” and “perinatal + adult” groups caused an increase in P16-positive and P21-positive hepatocytes after 26 or ~75 weeks compared to the respective control group (Figure 5 and Figure 6). Interestingly, administration of GW7647 did not further influence the number of P16-positive or P21-positive hepatocytes in the “adult only” and “perinatal + adult” groups of *Ppara*-null and *PPARA*-humanized mice whose expression of P16 and P21 were higher as compared to control wild-type mice (Figure 5 and Figure 6). 

### 3.6. Lower Liver CD4^+^ T Cells Are Associated with Reduced Production of Serum Cytokines

**Figure 7:**
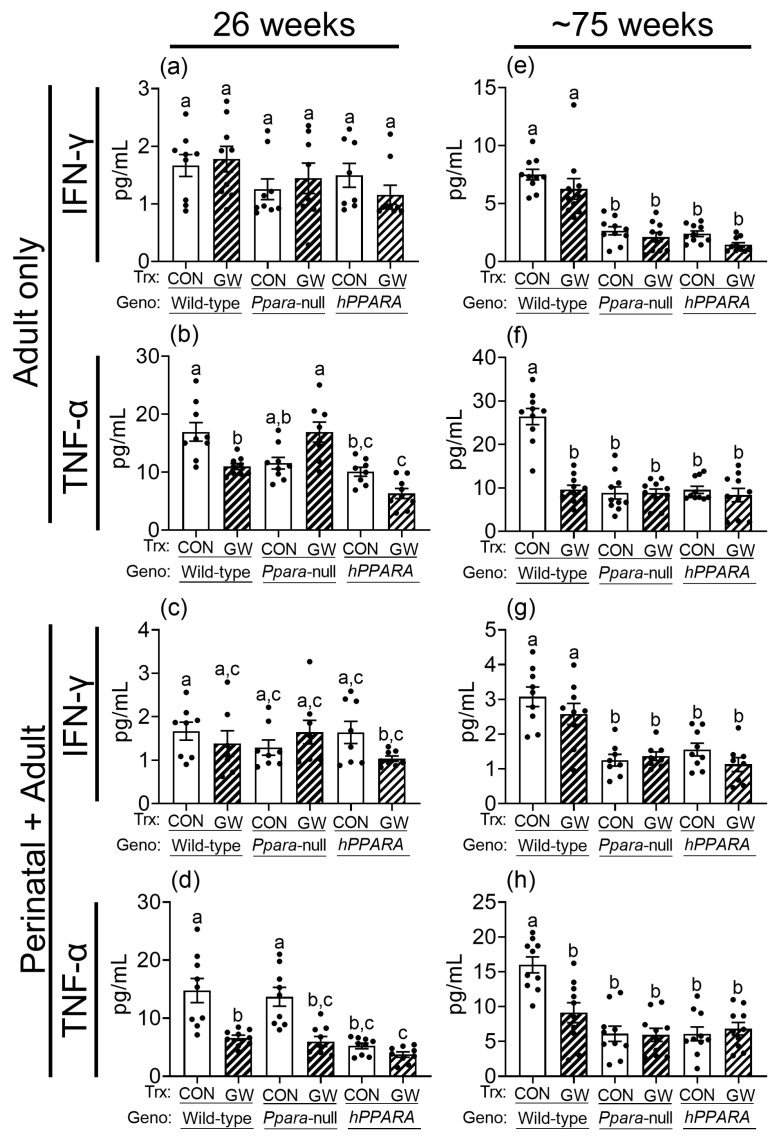
Average serum concentration of IFNγ (**a**,**c**,**e**,**g**), TNFα (**b**,**d**,**f**,**h**) in “adult only” and “perinatal + adult” groups of wild-type, *Ppara*-null, or *PPARA*-humanized mice with or without ligand activation of PPARα with GW7647 after 26 weeks (**left panels**) or ~75 weeks (**right panels**). Values represent the mean ± S.E.M. Groups with different superscript letters are significantly different at *p* ≤ 0.05. (One-way ANOVA followed by Tukey’s multiple comparisons test).

Since CD4^+^ T cells regulate the production of gamma interferon (IFN-γ) and tumor necrosis factor alpha (TNF-α), which have important roles in immune surveillance, these were measured in serum. Serum IFN-γ was similar between control wild-type, *Ppara*-null, and *PPARA*-humanized mice in both the “adult only” and “perinatal + adult” groups of control mice after 26 weeks (Figure 7). Serum IFNγ was lower in *Ppara*-null and *PPARA*-humanized mice in both the “adult only” and “perinatal + adult” groups compared to control wild-type mice after ~75 weeks (Figure 7). Ligand activation of PPARα with GW7647 did not influence serum IFNγ in either the “adult only” and “perinatal + adult” groups compared to wild-type controls after 26 or ~75 weeks (Figure 7). Serum TNFα was similar between control wild-type and *Ppara*-null mice in the “adult only” and “perinatal + adult” groups of mice after 26 weeks (Figure 7). By contrast, serum TNFα was lower in control *PPARA*-humanized mice compared to control wild-type mice in the “adult only” and “perinatal + adult” groups after 26 weeks (Figure 7). Serum TNFα was lower in control *Ppara*-null and *PPARA*-humanized mice compared to control wild-type mice in the “adult only” and “perinatal + adult” groups after ~75 weeks (Figure 7). Ligand activation of PPARα with GW7647 in wild-type mice decreased serum TNFα in both the “adult only” and “perinatal + adult” groups compared to wild-type controls after 26 or ~75 weeks (Figure 7). This effect did not occur in similarly treated *Ppara*-null mice in the “adult only” groups (Figure 7). Administration of GW7647 caused lower serum TNFα in *Ppara*-null mice in the “perinatal + adult” group, but this effect was not observed after ~75 weeks (Figure 7). Administration of GW7647 to *PPARA*-humanized mice had no effect on serum TNFα in both the “adult only” and “perinatal + adult” groups compared to controls after either 26 or ~75 weeks (Figure 7).

## 4. Discussion

Results from these studies extend our understanding of the role of metabolism in liver cancer. The use of wild-type, *Ppara*-null, or *PPARA*-humanized mice, coupled with chronic ligand activation of PPARα administered using different ages of mice (“adult only” and “perinatal + adult”) to initiate ligand activation of PPARα provided a platform to critically examine: (1) how liver metabolism adapts during PPARα agonist-induced liver carcinogenesis; (2) whether mouse or human PPARα modulate liver metabolism differentially during PPARα agonist-induced liver carcinogenesis; and (3) whether aging influenced either liver metabolism and/or species differences in PPARα agonist-induced liver carcinogenesis.

Chronic ligand activation of PPARα causes liver cancer in rodents [48]. However, *Ppara*-null mice are resistant to PPARα agonists-induced liver cancer demonstrating that PPARα mediates hepatocarcinogenesis resulting from exposure to these non-genotoxic rodent carcinogens [13,14,15,16,17]. Diminished liver carcinogenesis has also been observed in *PPARA*-humanized mice after chronic administration of PPARα agonists demonstrating the mechanism of species differences in liver cancer between rodents and humans [13,14,16]. These observations coupled with a larger body of evidence indicate that rodents can develop liver cancer after chronic administration of PPARα agonist whereas humans do not [5,9,10,11,21,22,23,49,50]. Results from the present studies show that PPARα-dependent effects increase fatty acids, triglycerides, and monounsaturated fatty acids in the liver metabolome in wild-type mice, and are not found in similarly treated *Ppara*-null mice. Interestingly, this is consistent with the finding that ligand activation of PPARα caused a small receptor-dependent increase in the concentration of hepatic linoleic acid after 26 weeks, but this change was not noted after ~75 weeks. Whether this change is causally related to, or associated with, PPARα agonist-induced hepatocarcinogenesis noted in the previous studies [13,14] is uncertain. As these PPARα-dependent effects were the same in both “adult only” and “perinatal + adult” groups compared to control suggests that this effect is not impacted by aging. However, it is worth noting that changes in liver linoleic acid were also noted in older control and GW7647-treated *Ppara*-null and *PPARA*-humanized mice, and the observed changes in liver linoleic acid were different than those observed in wild-type mice.

While there is strong evidence that PPARα is required to mediate liver carcinogenesis caused by chronic ligand activation of PPARα in rodents, there is equally convincing evidence that humans do not develop liver cancer following administration of PPARα agonists (reviewed in [5,9,10,11,21,22,49,50]). This is based on different types of studies ranging from retrospective and prospective studies showing no change in liver cancer in humans treated with PPARα ligands (e.g., fibrate drugs), to preclinical studies showing that *Ppara*-null and *PPARA*-humanized mice are refractory to liver carcinogenesis after long-term administration of PPARα agonists [5,9,13,14,15,16,21,22], and lack of increased proliferation of human hepatocytes in chimeric humanized mice [23]. Indeed, there is a consensus that ligand activation of PPARα can cause liver carcinogenesis in rodents but ligand activation of PPARα in humans does not [5,9,21,22]. However, it is critical to note that both *Ppara*-null and *PPARA*-humanized mice both develop liver tumors with or without administration of PPARα agonists but that the observed phenotype is different than that found in wild-type mice [13,14,15,24]. The present studies provide new evidence that may explain how liver carcinogenesis found in aged *Ppara*-null and *PPARA*-humanized mice is different than that which occurs by ligand activation of PPARα. In the *Ppara*-null and *PPARA*-humanized mice, there is an accumulation of hepatocellular lipids, leading to macrosteatosis (a form of hepatocellular toxicity) with consequent increased cellular proliferation and an increased risk of hepatocellular cancer [13,14,21,51]. This accumulation of fat is a consequence of the altered lipid metabolism occurring in the absence of PPARα expression.

It is well-established that PPARα regulates the expression of key gene products involved in metabolism including fatty acid binding, mitochondrial and peroxisomal fatty acid oxidation, ketogenesis, triglyceride turnover, gluconeogenesis, and bile synthesis/secretion [52,53]. Additionally, expression of PPARα is relatively high in the liver because lipids are extensively metabolized in this tissue [52,53]. This is consistent with the reduced constitutive expression of mitochondrial fatty acid metabolizing enzymes in the liver and accompanies an increase in serum and liver lipids in *Ppara*-null mice compared to wild-type controls [25,54]. Results from the present analyses demonstrated enhanced hepatic lipid accumulation with aging, which is similar to the phenotype noted in other studies in both *Ppara*-null and *PPARA*-humanized mice [13,14]. Accumulation of intracellular lipids can be oxidized to produce peroxidation products that can be mutagenic and carcinogenic [55]. Since no changes in hepatic 4-HNE and MDA concentration were detected in either *Ppara*-null and *PPARA*-humanized mice, this suggests that peroxidation of accumulated lipids does not appear to contribute to the mechanisms that cause liver tumors in *Ppara*-null and *PPARA*-humanized mice.

Results from these studies demonstrate for the first time that the concentration of linoleic acid is markedly higher in the liver of relatively older *Ppara*-null and *PPARA*-humanized mice compared to older wild-type mice. The higher concentration of liver linoleic acid was observed in both “adult only” and “perinatal + adult” groups compared to controls, and this effect was greatest after ~75 weeks. This effect is likely due in part to the aging of *Ppara*-null and *PPARA*-humanized mice compared to wild-type mice. Initiation of ligand administration during perinatal development of *Ppara*-null and *PPARA*-humanized mice (“perinatal + adult” groups) caused similar changes in hepatic linoleic acid concentration as those observed in the “adult only” groups. 

High levels of hepatic linoleic acid can selectively cause CD4^+^ T cell loss in the liver [45,47,56], and the antitumor T-cell response in liver carcinogenesis can be inhibited by high dietary linoleic acid [46]. Results from the present studies demonstrate a decrease in the number of hepatic CD4^+^ T cells in *Ppara*-null and *PPARA*-humanized mice that inversely correlated with the concentration of linoleic acid in the liver. Administration of GW7647 did not further impact this phenotype in either *Ppara*-null and *PPARA*-humanized mice, in both the “adult only” and “perinatal + adult” groups of mice. This suggests that the higher liver linoleic acid in *Ppara*-null and *PPARA*-humanized mice may cause a decrease in CD4^+^ T cells, consistent with past studies [45,47,56]. Furthermore, since CD4^+^ T cells are critical for tumor surveillance and suppression, this indicates that reduced CD4^+^ T cells could lead to abnormal cell signaling that causes liver tumorigenesis [56,57,58,59]. 

IFNγ and TNFα are known to mediate some antitumor effects of CD4^+^ T cells [60,61,62]. Thus, it is of interest to note that the serum concentrations of IFNγ and TNFα are significantly lower in *Ppara*-null and *PPARA*-humanized mice compared to wild-type controls consistent with the lower levels of CD4^+^ T cells found in these mice. These data collectively suggest that higher liver linoleic acid may cause CD4^+^ T cell loss and reduced expression of IFNγ and TNFα in the liver of *Ppara*-null and *PPARA*-humanized mice, and this mechanism may contribute to the age-related hepatocarcinogenesis observed in these mice. Administration of GW7647 did not further affect serum concentrations of IFNγ and TNFα compared to control *Ppara*-null and *PPARA*-humanized mice in both the “adult only” and “perinatal + adult” groups of mice after ~75 weeks. Combined, these data suggest that ligand activation of mouse PPARα is associated with lower CD4^+^ T cells in wild-type mice, and that this may contribute to reduced expression of TNFα (but not IFNγ) and less antitumor immune function in the liver. By contrast, genetic silencing of PPARα expression causes larger increases in liver linoleic acid over time, and this phenotype is accompanied with a reduced CD4^+^ T cell number in the liver, and lower serum IFNγ and TNFα compared to wild-type controls. Interestingly, this indicates that this phenotype observed in the absence of PPARα expression (*Ppara*-null mice), differs from that found in response to ligand activation of PPARα in wild-type mice. Moreover, the administration of GW7647 did not markedly alter this phenotype demonstrating that this ligand does not activate PPARα in *Ppara*-null mice. *PPARA*-humanized mice exhibit a similar phenotype to *Ppara*-null mice with higher liver linoleic acid over time accompanied with a reduced hepatic CD4^+^ T cell number and lower serum IFNγ and TNFα compared to wild-type controls, a phenotype not further impacted by administration of GW7647. Since GW7647 is a potent human PPARα agonist, these observations suggest that there is a species difference in the ability of the human PPARα to modulate the same pathways as the mouse PPARα.

CD4^+^ T cells have important roles in tumorigenesis because they help with turnover of senescent cells [57,63,64,65]. Aging is associated with an increase in senescence because of less surveillance of tumors provided by CD4^+^ T cells [57,63,64,65]. Based on the expression of P16 and P21, the present studies suggest that ligand activation caused a mouse PPARα-dependent increase in senescent liver cells. This effect may represent a new mechanism that contributes to PPARα agonist-induced mouse liver carcinogenesis mediated by enhanced hepatocyte proliferation accompanied with accumulation of more senescent liver cells. Importantly, the changes in this signaling in wild-type mice are markedly different than those found in *Ppara*-null and *PPARA*-humanized mice. The number of senescent cells in control *Ppara*-null and *PPARA*-humanized mouse liver was higher compared to control wild-type, and this effect was greater in older *Ppara*-null and *PPARA*-humanized mice compared to control wild-type mice. The increase in senescent cells in *Ppara*-null and *PPARA*-humanized mice could be due to the impaired function of CD4^+^ T cells as a result of aging. The changes in liver cell senescence in relatively older *Ppara*-null and *PPARA*-humanized mice were comparable to liver cell senescence in wild-type mice observed in response to ligand activation of PPARα, independent of GW7647 administration and age. As cellular senescence is one mechanism involved in the regulation and suppression of tumor cells [64], the differential increase in senescent liver cells could contribute to the mechanisms mediating liver carcinogenesis observed in older *Ppara*-null and *PPARA*-humanized mouse liver [66,67]. The role of immunosurveillance activities of CD4^+^ T cells is best characterized in viral-dependent cancer models [56,57,58,59]. Thus, since hepatic macrosteatosis can cause hepatocyte cytotoxicity, enhanced inflammation, and regenerative proliferation, it remains possible that the reduction in CD4^+^ T cells noted is secondary to hepatic macrosteatosis. Alternatively, the changes in liver metabolism noted in the present studies may be influenced/reflected by hepatic enzymes that can also interact with other pathways (e.g., immune system and cellular metabolism) and contribute to disease progression [68].

## 5. Conclusions

Collectively, these findings indicate that PPARα agonists cause liver cancer in mice through a mechanism that is associated with PPARα-dependent modulation of lipid metabolism including modest accumulation of linoleic acid, reduced hepatic CD4^+^ T cells, increased cellular senescence, and lowered serum cytokines. These studies also demonstrate a novel, alternative mechanism for carcinogenesis related to the accumulation of hepatic linoleic acid in *Ppara*-null and *PPARA*-humanized mice, an effect related to the loss of the PPARα regulation of lipid metabolism in hepatocytes. While no loss-of-function mutations of *PPARA* have been reported, results from these studies suggest that mutant, less functional PPARα could be a risk factor for liver cancer due to dysfunctional regulation of linoleic acid metabolism, reduced CD4^+^ T cell presence and function, and increased hepatocyte senescence. Results from these studies also indicate that liver tumors found in older *Ppara*-null and *PPARA*-humanized mice are mediated by the accumulation of liver linoleic acid that impairs CD4^+^ T cells in the liver and causes age-related accumulation of senescent liver cells. *Ppara*-null and *PPARA*-humanized mouse models remain valuable models to study the mechanisms of PPARα-agonist induced hepatocarcinogenesis, but background liver tumorigenesis must be factored into the interpretation of the results of such studies.

## Data Availability

The data presented in this study are available on request from the corresponding author. The data are not publicly available due to the author’s preference.

## Supplementary Materials

The following supporting information can be downloaded at: https://www.mdpi.com/article/10.3390/metabo13080936/s1, Figure S1: Schematic of two treatment paradigms; Figure S2: Representative 600 MHz ^1^H-NMR spectrum of lipophilic mouse liver extract; Figure S3: Relative levels of fatty acids (a and e), triglycerides (b and f), unsaturated fatty acids (UFA; c and g), or polyunsaturated fatty acids (PUFA; d and h) in lipophilic liver extracts from “perinatal + adult” groups of wild-type, *Ppara*-null or *PPARA*-humanized mice with or without ligand activation of PPARα after 26 weeks (a-d) or ~75 weeks (e–h); Figure S4: Relative average concentration of ω-3 fatty acids (a, c, e, g) or docosahexaenoic acid (DHA; b, d, f, h), in lipophilic liver extracts from “adult only” and “perinatal + adult” groups of wild-type, *Ppara*-null, or *PPARA*-humanized mice with or without ligand activation of PPARα with GW7647 after 26 weeks (left panels) or ~75 weeks (right panels); Figure S5: Determining liver linoleic acid with GC-MS in liver from “adult only” (a) and “perinatal + adult” (b) groups of wild-type, *Ppara*-null, or *PPARA*-humanized mice with or without ligand activation of PPARα after ~75 weeks; Figure S6: Correlation between liver linoleic acid concentration and the number of CD4^+^ T cells in liver of in “adult only” (upper panels) and “perinatal + adult” (lower panels) groups of wild-type, *Ppara*-null, or *PPARA*-humanized mice after 26 weeks (left panels) or ~75 weeks (right panels); Tables S1–S4: Summary of other significantly changed liver lipids in mice of groups.
