# Supplementary material for: Accumulation of Linoleic Acid by Altered Peroxisome Proliferator-Activated Receptor-α Signaling Is Associated with Age-Dependent Hepatocarcinogenesis in Ppara Transgenic Mice
Source: Metabolites. 2023 Aug 10;13(8):936. doi: 10.3390/metabo13080936 (PMC10456914; doi:10.3390/metabo13080936)
Supplement: Supplementary file 1 [file metabolites-13-00936-s001.zip › metabolites-2524933-supplementary-figures.pdf]

Figure S1

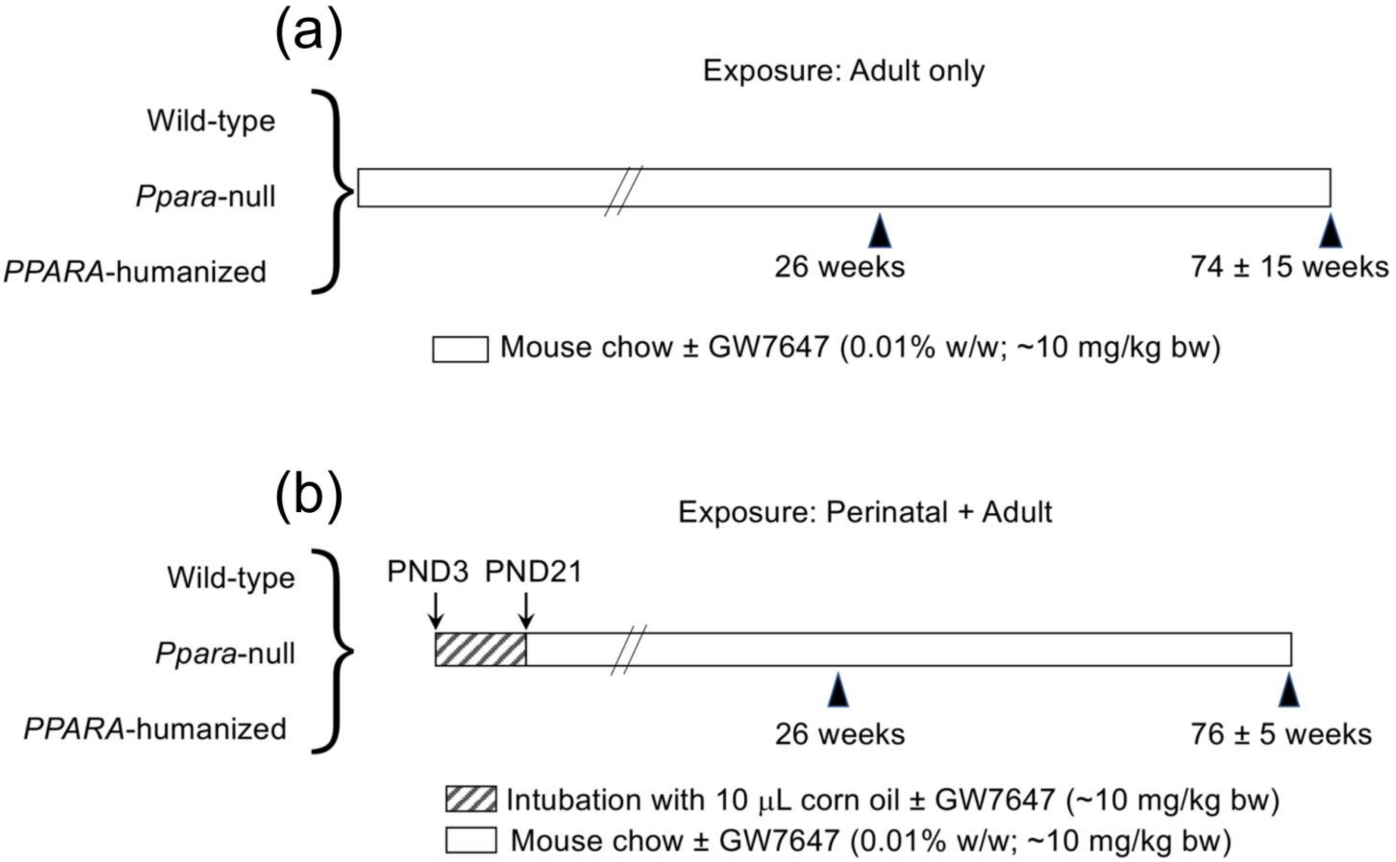

Figure S1. Schematic of two treatment paradigms. "Adult only" (Upper panel, a): Adult male wild-type, *Ppara*-null or *PPARA*-humanized mice were fed either a control diet or one containing 0.001% GW7647 for either 26 weeks or ~75 weeks, and tissues examined at each time point. "Perinatal + adult" (Lower panel, b): Postnatal day 3 male wild-type, *Ppara*-null or *PPARA*-humanized mouse neonates were gavaged with either corn oil (vehicle control) or GW7647 (10 mg/kg) until weaning. Cohorts of neonatal pups were weaned at the age of 3 weeks. After weaning, mice were then fed either a control diet or one containing 0.001% GW7647 to provide for examination after a total of either 26 weeks or ~75 weeks administration.

Figure S2

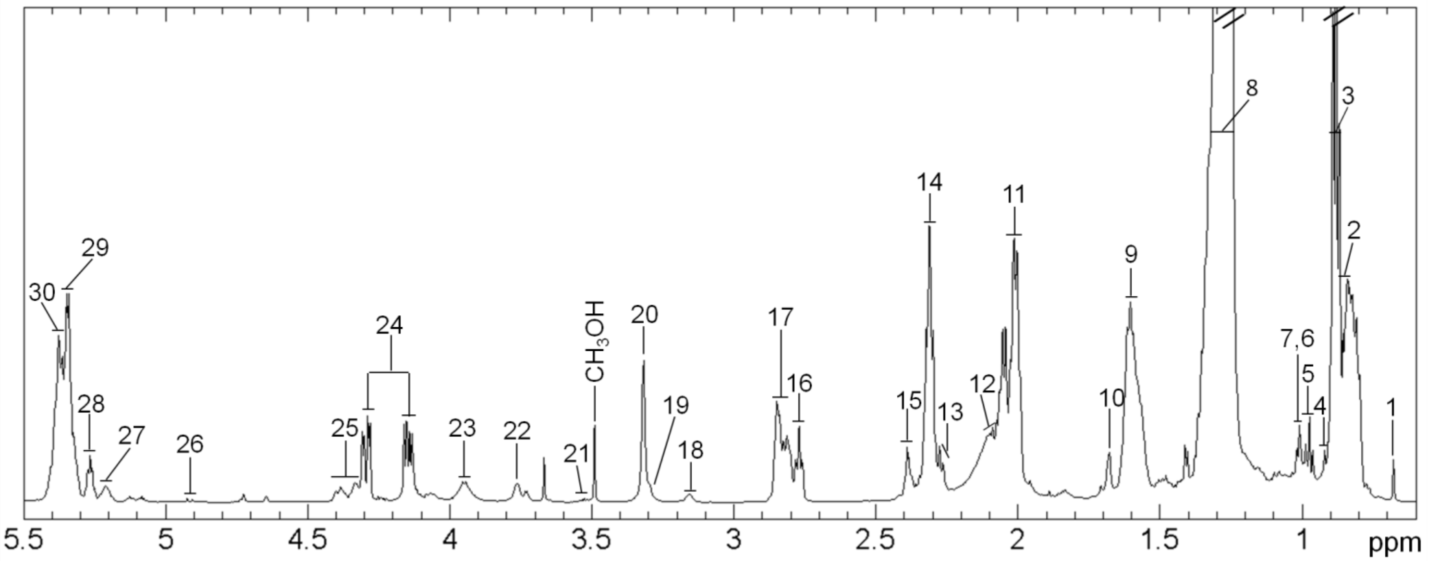

Figure S2. Representative 600 MHz ^{1}H-NMR spectrum of lipophilic mouse liver extract. Keys: 1. Total cholesterol ($C_{18}\mathbf{H}_3$), s; 2. Total cholesterol ($C_{26}\mathbf{H}_3$, $C_{27}\mathbf{H}_3$), d; 3. Fatty acid residues (R-C$\mathbf{H}_3$), t; 4. Total cholesterol ($C_{21}\mathbf{H}_3$), d; 5. Fatty acid residues (ω-3, R-C$\mathbf{H}_3$ of DHA, EPA, ALA, R-CH=CH-CH_2-C$\mathbf{H}_3$), t; 6. Free cholesterol ($C_{19}\mathbf{H}_3$), s; 7. Esterified cholesterol ($C_{19}\mathbf{H}_3$), s; 8. Fatty acid residues ((-C$\mathbf{H}_2$-)$_n$); 9. Fatty acid residues (R-C$\mathbf{H}_2$-CH_2-COO-); 10. Fatty acid residues (β-C$\mathbf{H}_2$ of ARA, EPA; -CH=CH-CH_2-C$\mathbf{H}_2$-CH_2-COO-); 11. Fatty acid residues (-C$\mathbf{H}_2$-CH=CH-(CH_2-CH=CH-)$_n$C$\mathbf{H}_2$-); 12. Fatty acid residues (γ-C$\mathbf{H}_2$ of ARA, EPA; -CH=CH-C$\mathbf{H}_2$-CH_2-CH_2-COO); 13. Monoglycerides (FA, RH-C$\mathbf{H}_2$-CO-O-C_2); 14. Fatty acid residues (-CO-C$\mathbf{H}_2$); 15. Fatty acid (α and β-C$\mathbf{H}_2$ of DHA, -CH=CH-C$\mathbf{H}_2$-C$\mathbf{H}_2$-COO); 16. Fatty acid residues (-CH=CH-C$\mathbf{H}_2$-CH=CH- of linoleic acid); 17. FA, PUFA (-CH=CH-C$\mathbf{H}_2$-(CH=CH-CH_2-)n); 18. Phosphatidylethanolamine (-C$\mathbf{H}_2$-NH_2); 19. Sphingomyelin (-CH_2-N-(C$\mathbf{H}_3$)$_3$); 20. Phosphatidylcholine (-CH_2-N-(C$\mathbf{H}_3$)$_3$); 21. Cholesterol (-C_3**H**-OH); 22. Phosphatidylcholine (-N-C$\mathbf{H}_2$-); 23. Glycerophospholipids backbone ($C_3\mathbf{H}_2$); 24. Triglycerides (C_1**H** and C_3**H** of glycerol); 25. Phosphatidylcholine (-P-O-C$\mathbf{H}_2$-); 26. Esterified cholesterol (-C_3**H**-OH); 27. Glycerophospholipid backbone (C_2**H**); 28. Triglycerides (C_2**H** of glycerol); 29. Fatty acid residues (-C**H**=C**H**-); 30. Cholesterol (C_6**H**). DHA, docosahexaenoic acid; EPA, eicosapentaenoic acid; ALA, alpha-linolenic acid; ARA, arachidonic acid.

Figure S3

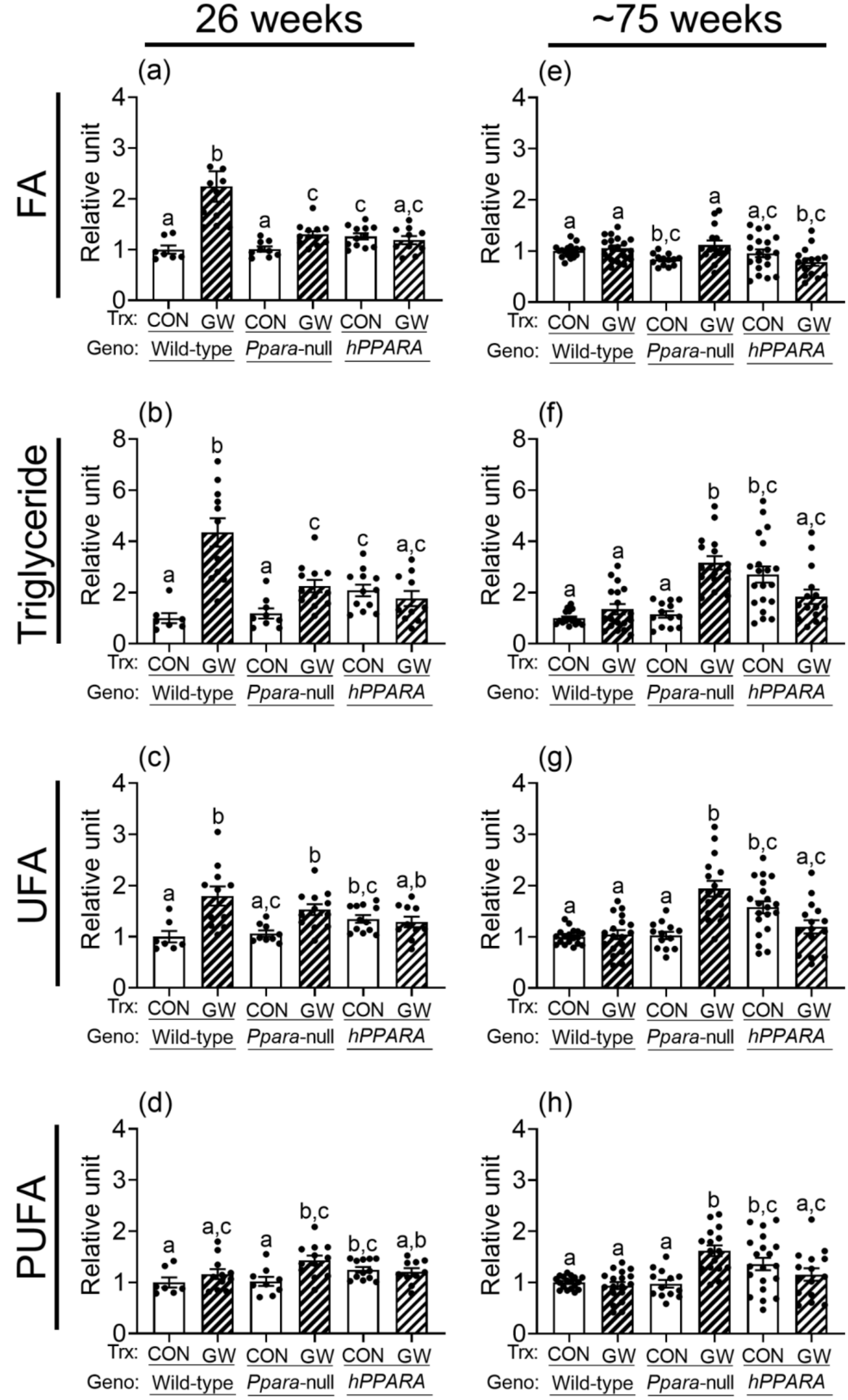

Figure S3. Relative levels of fatty acids (a and e), triglycerides (b and f), unsaturated fatty acids (UFA; c and g), or polyunsaturated fatty acids (PUFA; d and h) in lipophilic liver extracts from "perinatal + adult" groups of wild-type, *Ppara*-null or *PPARA*-humanized mice with or without ligand activation of PPARα after 26 weeks (a-d) or ~75 weeks (e-h). Fatty acid residues (R-C**H**$_3$), triglycerides (C$_1$**H** and C$_3$**H** of glycerol), unsaturated fatty acid (UFA) residues (-C**H**=C**H**-), polyunsaturated fatty acid (PUFA) residues (-CH=CH-C**H**$_2$-(CH=CH-CH$_2$-)n). The relative average amount of lipids in the liver of mice with different treatments was normalized to wild-type control and represents the fold change. Values represent the mean ± S.E.M. Groups with different superscript letters are significantly different at $P \leq 0.05$. (One-way ANOVA followed by Tukey's multiple comparisons test).

Figure S4

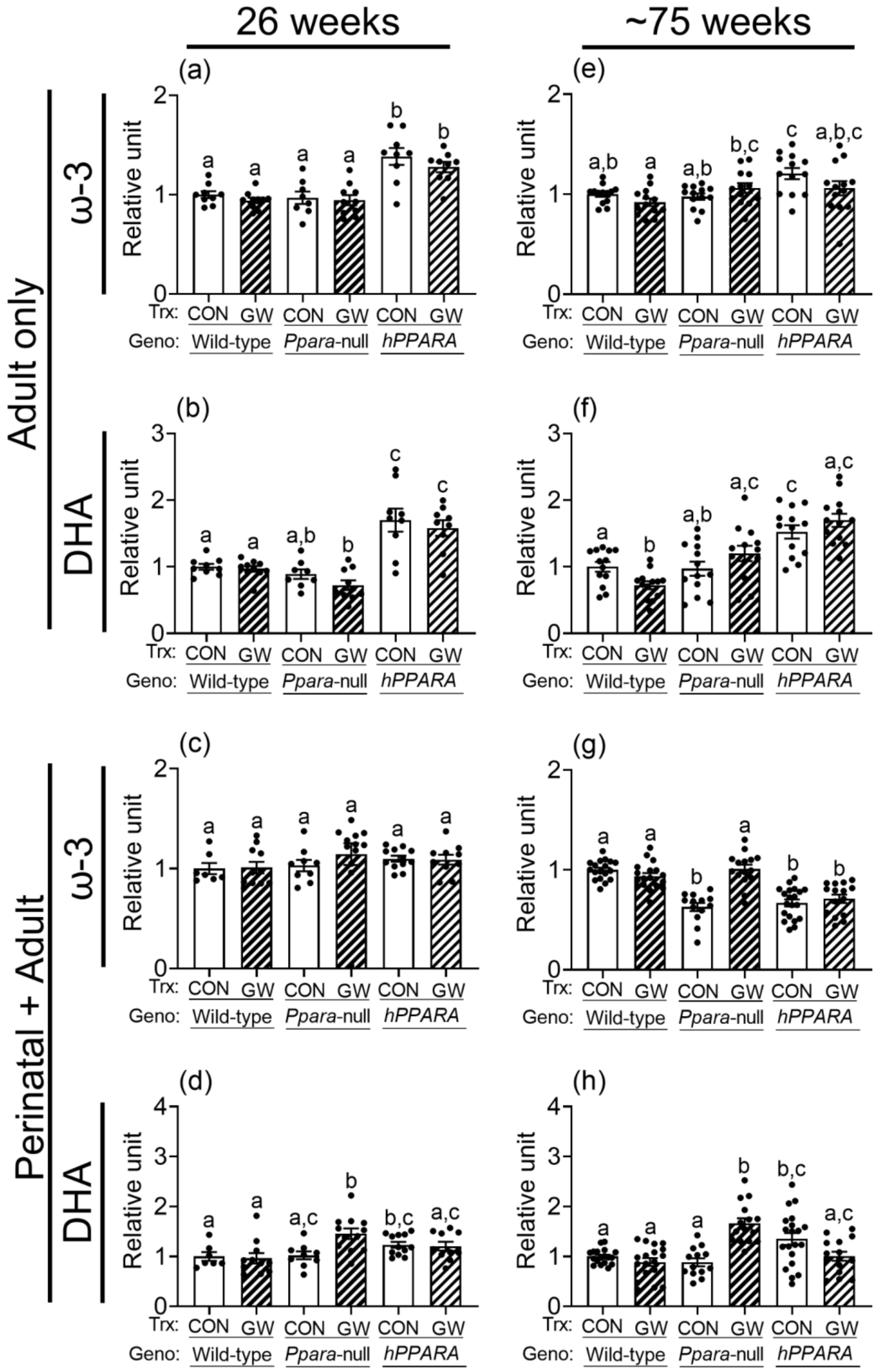

Figure S4. Relative average concentration of ω-3 fatty acids (a, c, e, g) or docosahexaenoic acid (DHA; b, d, f, h), in lipophilic liver extracts from “adult only” and “perinatal + adult” groups of wild-type, *Ppara*-null or *PPARA*-humanized mice with or without ligand activation of PPARα with GW7647 after 26 weeks (left panels) or ~75 weeks (right panels). The relative amount of lipids in liver was normalized to wild-type control and represents the fold change. Values represent the mean ± S.E.M. Groups with different superscript letters are significantly different at $P \leq 0.05$. (One-way ANOVA followed by Tukey’s multiple comparisons test).

Figure S5

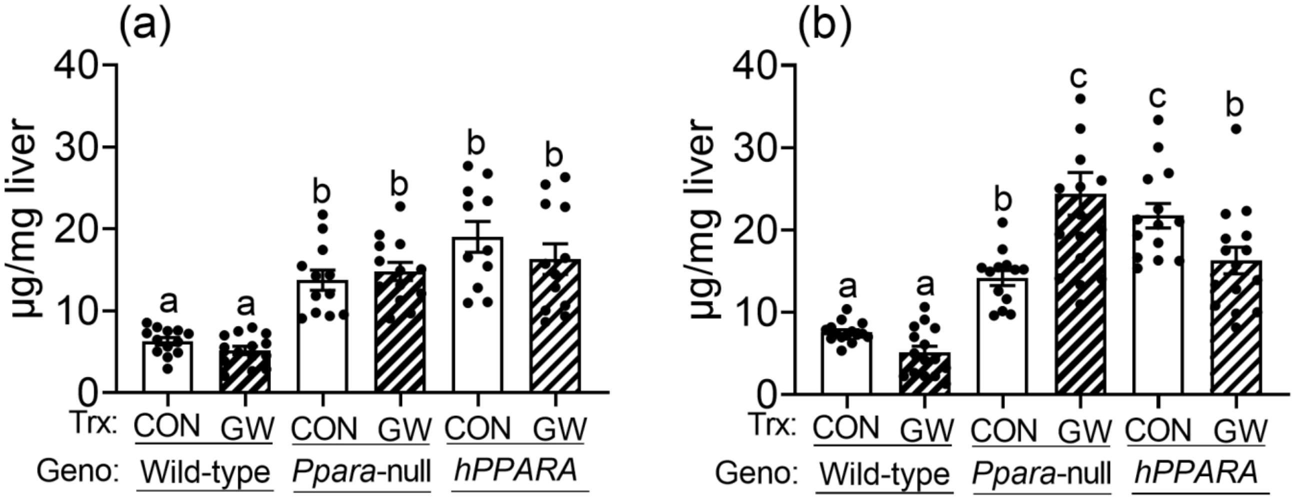

Figure S5. Determining liver linoleic acid with GC-MS in liver from “adult only” (a) and “perinatal + adult” (b) groups of wild-type, *Ppara*-null or *PPARA*-humanized mice with or without ligand activation of PPARα after ~75 weeks. Values represent the mean ± S.E.M. Groups with different superscript letters are significantly different at $P \leq 0.05$. (One-way ANOVA followed by Tukey’s multiple comparisons test).

Figure S6

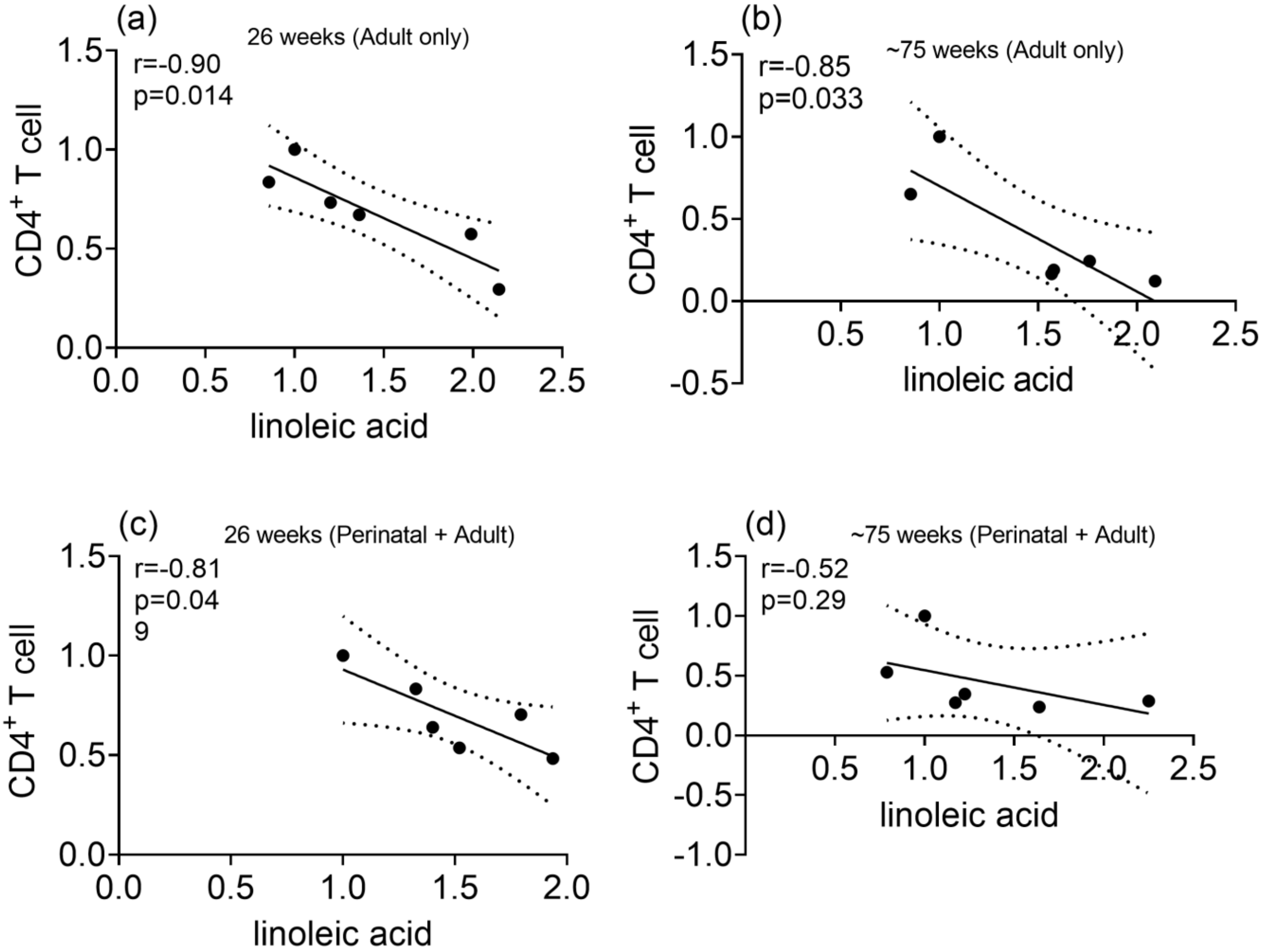

Figure S6. Correlation between liver linoleic acid concentration and the number of $CD4^+$ T cells in liver of in “adult only” (upper panels) and “perinatal + adult” (lower panels) groups of wild-type, *Ppara*-null or *PPARA*-humanized mice after 26 weeks (left panels) or ~75 weeks (right panels).
