# Supplementary material for: Accumulation of Linoleic Acid by Altered Peroxisome Proliferator-Activated Receptor-α Signaling Is Associated with Age-Dependent Hepatocarcinogenesis in Ppara Transgenic Mice
Source: Metabolites. 2023 Aug 10;13(8):936. doi: 10.3390/metabo13080936 (PMC10456914; doi:10.3390/metabo13080936)
Supplement: Supplementary file 1 [file metabolites-13-00936-s001.zip › metabolites-2524933-supplementary-tables.pdf]

Table S1. Summary of other significantly changed liver lipids in mice of groups, when compared with wild-type control or wild-type GW7647 treated group after 26 weeks administration (Adult only).

Residues of metabolites	26 weeks (adult)								
	B^A	C^A	D^A	E^A	F^A	C^B	D^B	E^B	F^B
1. Total cholesterol ($C_{18}\mathbf{H_3}$), s	–		+	–	–	+	+		
2. Total cholesterol ($C_{26}\mathbf{H_3}$, $C_{27}\mathbf{H_3}$), d									
4. Total cholesterol ($C_{21}\mathbf{H_3}$), d									
6. Free cholesterol ($C_{19}\mathbf{H_3}$), s	–			–	–	+	+		
7. Esterified cholesterol ($C_{19}\mathbf{H_3}$), s	–			–	–	+	+	+	
9. Fatty acid -C$\mathbf{H_2}$- CH_2-COO	+		+	+	+	–	–	–	
10. Fatty acid -CH=CH-C$\mathbf{H_2}$-CH_2-CH_2-COO	–		+	–	–	+	+	+	
11. Fatty acid -C$\mathbf{H_2}$-CH=CH-(CH_2-CH=CH-) $_n$C$\mathbf{H_2}$	+			+	+	–	–	–	–
12. Fatty acid (γ-C$\mathbf{H_2}$ of ARA, EPA) -CH=CH-C$\mathbf{H_2}$-CH_2-CH_2-COO			–						
13. Monoglycerides (FA, RH-C$\mathbf{H_2}$-CO-O-C_2)	–			–	–	+	+		–
14. Fatty acid –CO-C$\mathbf{H_2}$	+		–	+	+	–	–		
18. Phophatidylethanolamine -C$\mathbf{H_2}$-NH_2	–			–	–	+	+		–
19. Sphingomyelin -CH_2-N-(C$\mathbf{H_3}$)$_3$	–			–	–	+	+		
20. Phosphatidylcholine -CH_2-N-(C$\mathbf{H_3}$)$_3$	–		–	–	–	+	+		–
21. Cholesterol 3-C**H**OH									
22. Phosphatidylcholine -N-C$\mathbf{H_2}$-	–			–	–	+	+		–
23. Total phospholipids, glycerol ($C_3\mathbf{H_2}$)	–		–	–	–	+	+		–
25. Phosphatidylcholine -PO-C$\mathbf{H_2}$-	–		–	–	–	+			–
26. Esterified cholesterol 3-C**H**OH	–		–	–	–	+		+	
27. DG-2, Glycerophospholipid backbone 2-C**H**	–			–	–	+			–
30. Cholesterol C_6**H**	–			–	–	+	+	+	

A, control, wild-type mice without treatment; B group, wild-type mice with GW7647 treatment; C group, *Ppara*-null mice without treatment; D group, *Ppara*-null mice with GW7647 treatment; E group, *hPPARA* mice without treatmen; F group, *hPPARA* mice with GW7647 treatment. B^A, C^A, D^A, E^A, F^A, corresponding groups compared with wild-type control; C^B, D^B, E^B, F^B, corresponding groups compared with wild-type GW7647 treated groups. +, significantly increased; -, significantly decreased.

Table S2. Summary of other significantly changed liver lipids in mice of groups, when compared with wild-type control or wild-type GW7647 treated group after ~75 weeks administration (Adult only).

Residues of metabolites	Long term (adult)								
	B^A	C^A	D^A	E^A	F^A	C^B	D^B	E^B	F^B
1. Total cholesterol ($C_{18}\mathbf{H_3}$), s	+			–					
2. Total cholesterol ($C_{26}\mathbf{H_3}$, $C_{27}\mathbf{H_3}$), d									
4. Total cholesterol ($C_{21}\mathbf{H_3}$), d									
6. Free cholesterol ($C_{19}\mathbf{H_3}$), s				–					
7. Esterified cholesterol ($C_{19}\mathbf{H_3}$), s	–			–	–	+	+	+	+
9. Fatty acid -C**H₂**- CH₂-COO	+			+	+			+	
10. Fatty acid -CH=CH-C**H₂**-CH₂-CH₂-COO	+					+		+	
11. Fatty acid -C**H₂**-CH=CH-(CH₂-CH=CH-) ₙC**H₂**				+	+				
12. Fatty acid (γ-C**H₂** of ARA, EPA) -CH=CH-C**H₂**-CH₂-CH₂-COO									
13. Monoglycerides (FA, RH-C**H₂**-CO-O-C₂)	–			–					
14. Fatty acid –CO-C**H₂**				+	+		+	+	
18. Phophatidylethanolamine -C**H₂**-NH₂									
19. Sphingomyelin -CH₂-N-(C**H₃**)₃									
20. Phosphatidylcholine -CH₂-N-(C**H₃**)₃									
21. Cholesterol 3-C**H**OH									
22. Phosphatidylcholine -N-C**H₂**-									
23. Total phospholipids, glycerol ($C_3\mathbf{H_2}$)									
25. Phosphatidylcholine -PO-C**H₂**-									
26. Esterified cholesterol 3-C**H**OH	–			–	–	+	+	+	+
27. DG-2, Glycerophospholipid backbone 2-C**H**	+					–	–	–	
30. Cholesterol $C_6\mathbf{H}$									

A, control, wild-type mice without treatment; B group, wild-type mice with GW7647 treatment; C group, *Ppara*-null mice without treatment; D group, *Ppara*-null mice with GW7647 treatment; E group, *hPPARA* mice without treatmen; F group, *hPPARA* mice with GW7647 treatment. B^A, C^A, D^A, E^A, F^A, corresponding groups compared with wild-type control; C^B, D^B, E^B, F^B, corresponding groups compared with wild-type GW7647 treated groups. +, significantly increased; -, significantly decreased.

Table S3. Summary of other significantly changed liver lipids in mice of groups, when compared with wild-type control or wild-type GW7647 treated group after 26 weeks administration (Perinatal + Adult).

Residues of metabolites	26 weeks (perinatal)								
	B^A	C^A	D^A	E^A	F^A	C^B	D^B	E^B	F^B
1. Total cholesterol ($C_{18}\mathbf{H}_3$), s	–			–		+	+		
2. Total cholesterol ($C_{26}\mathbf{H}_3$, $C_{27}\mathbf{H}_3$), d									
4. Total cholesterol ($C_{21}\mathbf{H}_3$), d									
6. Free cholesterol ($C_{19}\mathbf{H}_3$), s	–		–	–		+	+	+	+
7. Esterified cholesterol ($C_{19}\mathbf{H}_3$), s	–		–	–		+	+	+	+
9. Fatty acid -C**H**$_2$- CH$_2$-COO	+		+	+		–	–	–	–
10. Fatty acid -CH=CH-C**H**$_2$-CH$_2$-CH$_2$-COO	–		–	–		+	+	+	+
11. Fatty acid -C**H**$_2$-CH=CH-(CH$_2$-CH=CH-) $_n$C**H**$_2$	+			+		–	–	–	–
12. Fatty acid (γ-C**H**$_2$ of ARA, EPA) -CH=CH-C**H**$_2$-CH$_2$-CH$_2$-COO	–					+	+	+	+
13. Monoglycerides (FA, RH-C**H**$_2$-CO-O-C$_2$)	–		–	–		+		+	+
14. Fatty acid –CO-C**H**$_2$	+		+	+		–	–	–	–
18. Phophatidylethanolamine -C**H**$_2$-NH$_2$	–		–	–		+			
19. Sphingomyelin -CH$_2$-N-(C**H**$_3$)$_3$	–		–			+			
20. Phosphatidylcholine -CH$_2$-N-(C**H**$_3$)$_3$	–		–	–		+		+	+
21. Cholesterol 3-C**H**OH									
22. Phosphatidylcholine -N-C**H**$_2$-	–		–	–		+			
23. Total phospholipids, glycerol (C$_3$**H**$_2$)	–		–	–		+	+		+
25. Phosphatidylcholine -PO-C**H**$_2$-	–		–	–		+		+	+
26. Esterified cholesterol 3-C**H**OH	–		–	–		+		+	+
27. DG-2, Glycerophospholipid backbone 2-C**H**	–		–	–		+		+	
30. Cholesterol C$_6$**H**	–		+	+		+	+	+	+

A, control, wild-type mice without treatment; B group, wild-type mice with GW7647 treatment; C group, *Ppara*-null mice without treatment; D group, *Ppara*-null mice with GW7647 treatment; E group, *hPPARA* mice without treatmen; F group, *hPPARA* mice with GW7647 treatment. B^A, C^A, D^A, E^A, F^A, corresponding groups compared with wild-type control; C^B, D^B, E^B, F^B, corresponding groups compared with wild-type GW7647 treated groups. +, significantly increased; -, significantly decreased.

Table S4. Summary of other significantly changed liver lipids in mice of groups, when compared with wild-type control or wild-type GW7647 treated group after ~75 weeks administration (Perinatal + Adult).

Residues of metabolites	Long term (perinatal)								
	B^A	C^A	D^A	E^A	F^A	C^B	D^B	E^B	F^B
1. Total cholesterol ($C_{18}\mathbf{H_3}$), s		+			+				
2. Total cholesterol ($C_{26}\mathbf{H_3}$, $C_{27}\mathbf{H_3}$), d									
4. Total cholesterol ($C_{21}\mathbf{H_3}$), d									
6. Free cholesterol ($C_{19}\mathbf{H_3}$), s									
7. Esterified cholesterol ($C_{19}\mathbf{H_3}$), s	–	+				+	+	+	
9. Fatty acid -C**H₂**- CH₂-COO		+	+	+			+	+	
10. Fatty acid -CH=CH-C**H₂**-CH₂-CH₂-COO						+	+	+	
11. Fatty acid -C**H₂**-CH=CH-(CH₂-CH=CH-) $_n$C**H₂**	+		+						–
12. Fatty acid (γ-C**H₂** of ARA, EPA) -CH=CH-C**H₂**-CH₂-CH₂-COO			+				+		
13. Monoglycerides (FA, RH-C**H₂**-CO-O-C_2)									–
14. Fatty acid –CO-C**H₂**			+	+	+		+	+	
18. Phophatidylethanolamine -C**H₂**-NH₂									–
19. Sphingomyelin -CH₂-N-(C**H₃**)₃						+			
20. Phosphatidylcholine -CH₂-N-(C**H₃**)₃		+		+	+				–
21. Cholesterol 3-C**H**OH									
22. Phosphatidylcholine -N-C**H₂**-					+				–
23. Total phospholipids, glycerol ($C_3\mathbf{H_2}$)		–	–		+				–
25. Phosphatidylcholine -PO-C**H₂**-					+			+	–
26. Esterified cholesterol 3-C**H**OH	–	+				+	+	+	
27. DG-2, Glycerophospholipid backbone 2-C**H**					+				–
30. Cholesterol $C_6\mathbf{H}$		+	+	+	+	+	+	+	+

A, control, wild-type mice without treatment; B group, wild-type mice with GW7647 treatment; C group, *Ppara*-null mice without treatment; D group, *Ppara*-null mice with GW7647 treatment; E group, *hPPARA* mice without treatmen; F group, *hPPARA* mice with GW7647 treatment. B^A, C^A, D^A, E^A, F^A, corresponding groups compared with wild-type control; C^B, D^B, E^B, F^B, corresponding groups compared with wild-type GW7647 treated groups. +, significantly increased; -, significantly decreased.
